# A Central Role for *GRB10* in Regulation of Islet Function in Man

**DOI:** 10.1371/journal.pgen.1004235

**Published:** 2014-04-03

**Authors:** Inga Prokopenko, Wenny Poon, Reedik Mägi, Rashmi Prasad B, S. Albert Salehi, Peter Almgren, Peter Osmark, Nabila Bouatia-Naji, Nils Wierup, Tove Fall, Alena Stančáková, Adam Barker, Vasiliki Lagou, Clive Osmond, Weijia Xie, Jari Lahti, Anne U. Jackson, Yu-Ching Cheng, Jie Liu, Jeffrey R. O'Connell, Paul A. Blomstedt, Joao Fadista, Sami Alkayyali, Tasnim Dayeh, Emma Ahlqvist, Jalal Taneera, Cecile Lecoeur, Ashish Kumar, Ola Hansson, Karin Hansson, Benjamin F. Voight, Hyun Min Kang, Claire Levy-Marchal, Vincent Vatin, Aarno Palotie, Ann-Christine Syvänen, Andrea Mari, Michael N. Weedon, Ruth J. F. Loos, Ken K. Ong, Peter Nilsson, Bo Isomaa, Tiinamaija Tuomi, Nicholas J. Wareham, Michael Stumvoll, Elisabeth Widen, Timo A. Lakka, Claudia Langenberg, Anke Tönjes, Rainer Rauramaa, Johanna Kuusisto, Timothy M. Frayling, Philippe Froguel, Mark Walker, Johan G. Eriksson, Charlotte Ling, Peter Kovacs, Erik Ingelsson, Mark I. McCarthy, Alan R. Shuldiner, Kristi D. Silver, Markku Laakso, Leif Groop, Valeriya Lyssenko

**Affiliations:** 1 Oxford Centre for Diabetes, Endocrinology and Metabolism, University of Oxford, Oxford, United Kingdom; 2 Wellcome Trust Centre for Human Genetics, University of Oxford, Oxford, United Kingdom; 3 Department of Genomics of Common Disease, School of Public Health, Imperial College London, Hammersmith Hospital, London, United Kingdom; 4 Department of Clinical Science, Diabetes & Endocrinology, Lund University Diabetes Centre, Malmö, Sweden; 5 Estonian Genome Center, University of Tartu, Tartu, Estonia; 6 University of Lille Nord de France, Lille, France; 7 CNRS UMR8199, Institut Pasteur de Lille, Lille, France; 8 INSERM U970, Paris Cardiovascular Research Center PARCC, Paris, France; 9 Department of Clinical Science, Neuroendocrine Cell Biology, Lund University Diabetes Centre, Malmö, Sweden; 10 Department of Medical Sciences, Molecular Epidemiology and Science for Life Laboratory, Uppsala University, Uppsala, Sweden; 11 Department of Medicine, University of Eastern Finland and Kuopio University Hospital, Kuopio, Finland; 12 MRC Epidemiology Unit, Institute of Metabolic Science, University of Cambridge, Cambridge, United Kingdom; 13 MRC Lifecourse Epidemiology Unit, University of Southampton, Southampton, United Kingdom; 14 Peninsula College of Medicine and Dentistry, University of Exeter, Exeter, United Kingdom; 15 Institute of Behavioural Sciences, University of Helsinki, Helsinki, Finland; 16 Folkhälsan Research Centre, Helsinki, Finland; 17 Department of Biostatistics and Center for Statistical Genetics, University of Michigan, Ann Arbor, Michigan, United States of America; 18 Division of Endocrinology Diabetes and Nutrition, Department of Medicine, University of Maryland School of Medicine, Baltimore, Maryland, United States of America; 19 Department of Chronic Disease Prevention, National Institute for Health and Welfare, Helsinki, Finland; 20 Department of Mathematics, Åbo Akademi University, Turku, Finland; 21 Epigenetics and Diabetes Unit, Department of Clinical Sciences, Lund University, CRC, Scania University Hospital, Malmö, Sweden; 22 Swiss Tropical and Public Health Institute, University of Basel, Basel, Switzerland; 23 Department of Pharmacology and Department of Genetics, University of Pennsylvania Perelman School of Medicine, Philadelphia, Pennsylvania, United States of America; 24 INSERM - Institut de Santé Publique, Paris, France; 25 INSERM CIC EC 05, Hôpital Robert Debré, Paris, France; 26 Institute for Molecular Medicine Finland (FIMM), University of Helsinki, Helsinki, Finland; 27 Wellcome Trust Sanger Institute, Wellcome Trust Genome Campus, Cambridge, United Kingdom; 28 Department of Medicine, Helsinki University Central Hospital, Helsinki, Finland; 29 Program in Medical and Population Genetics and Genetics Analysis Platform, The Broad Institute of Massachusetts Institute of Technology and Harvard, Cambridge, Massachusettes, United States of America; 30 Molecular Medicine, Department of Medical Sciences, Science for Life Laboratory, Uppsala University, Uppsala, Sweden; 31 CNR Institute of Biomedical Engineering, Padova, Italy; 32 Department of Clinical Science, Internal Medicine, Skåne University Hospital Malmö, Malmö, Sweden; 33 Department of Social Service and Health Care, Jakobstad, Finland; 34 University of Leipzig, Department of Medicine, Leipzig, Germany; 35 University of Leipzig, IFB Adiposity Diseases, Leipzig, Germany; 36 Institute of Biomedicine/Physiology, University of Eastern Finland, Kuopio, Finland; 37 Kuopio Research Institute of Exercise Medicine, Kuopio, Finland; 38 Department of Clinical Physiology and Nuclear Medicine, Kuopio University Hospital, Kuopio, Finland; 39 Institute of Cellular Medicine, Newcastle University, Newcastle upon Tyne, United Kingdom; 40 Helsinki University, Department of General Practice and Primary Health Care, Helsinki, Finland; 41 Helsinki University Central Hospital, Unit of General Practice, Helsinki, Finland; 42 Oxford NIHR Biomedical Research Centre, Churchill Hospital, Oxford, United Kindom; 43 Baltimore Geriatric Research, Education and Clinical Center, Baltimore, Maryland, United States of America; 44 Steno Diabetes Center A/S, Gentofte, Denmark; Dartmouth College, United States of America

## Abstract

Variants in the growth factor receptor-bound protein 10 (*GRB10*) gene were in a GWAS meta-analysis associated with reduced glucose-stimulated insulin secretion and increased risk of type 2 diabetes (T2D) if inherited from the father, but inexplicably reduced fasting glucose when inherited from the mother. GRB10 is a negative regulator of insulin signaling and imprinted in a parent-of-origin fashion in different tissues. *GRB10* knock-down in human pancreatic islets showed reduced insulin and glucagon secretion, which together with changes in insulin sensitivity may explain the paradoxical reduction of glucose despite a decrease in insulin secretion. Together, these findings suggest that tissue-specific methylation and possibly imprinting of *GRB10* can influence glucose metabolism and contribute to T2D pathogenesis. The data also emphasize the need in genetic studies to consider whether risk alleles are inherited from the mother or the father.

## Author Summary

In this paper, we report the first large genome-wide association study in man for glucose-stimulated insulin secretion (GSIS) indices during an oral glucose tolerance test. We identify seven genetic loci and provide effects on GSIS for all previously reported glycemic traits and obesity genetic loci in a large-scale sample. We observe paradoxical effects of genetic variants in the growth factor receptor-bound protein 10 (*GRB10*) gene yielding both reduced GSIS and reduced fasting plasma glucose concentrations, specifically showing a parent-of-origin effect of *GRB10* on lower fasting plasma glucose and enhanced insulin sensitivity for maternal and elevated glucose and decreased insulin sensitivity for paternal transmissions of the risk allele. We also observe tissue-specific differences in DNA methylation and allelic imbalance in expression of *GRB10* in human pancreatic islets. We further disrupt *GRB10* by shRNA in human islets, showing reduction of both insulin and glucagon expression and secretion. In conclusion, we provide evidence for complex regulation of *GRB10* in human islets. Our data suggest that tissue-specific methylation and imprinting of *GRB10* can influence glucose metabolism and contribute to T2D pathogenesis. The data also emphasize the need in genetic studies to consider whether risk alleles are inherited from the mother or the father.

## Introduction

Type 2 diabetes (T2D) is a multifactorial polygenic disease, in which genes interact with environmental and genetic factors to unmask the disease. To date, genome-wide association studies (GWAS) have identified more than 65 variants increasing risk of T2D [1]–[8]. Many of the identified variants seem to influence the capacity of β-cells to cope with increased insulin demands imposed by insulin resistance [9], [10]. Despite this, no GWAS to date has explored the extent to which common genetic variants influence dynamic measures of insulin secretion and action. Therefore, we have here performed the first large-scale meta-analysis for glucose-stimulated insulin secretion (GSIS) during an oral glucose tolerance test (OGTT). Association analyses included a GWAS-based discovery stage and a replication stage using a custom-designed iSelect CardioMetabochip array containing 93,896 SNPs overlapping with the discovery GWAS imputed from the HapMap2 reference panel in up to 10,831 non-diabetic individuals. We identified variants in the growth factor receptor-bound protein 10 (*GRB10*) gene (NCBI Gene ID: 2887) to be associated with impaired β-cell function at a genome-wide significant level (*p*<5×10^−8^). Since *GRB10* has been shown to have parent-of-origin specific effects on expression in various tissues [11], [12], we investigated the transmission patterns of the risk alleles and their effects on insulin and glucose levels during an OGTT, risk for T2D, expression of *GRB10* and methylation status in human pancreatic islets, as well as evaluated the effects on islet function through disruption of *GRB10* in human pancreatic islets.

## Results

### Meta-Analysis of GWAS and CardioMetabochip Studies for Insulin Secretion and Action during an OGTT

**Figure 1:**
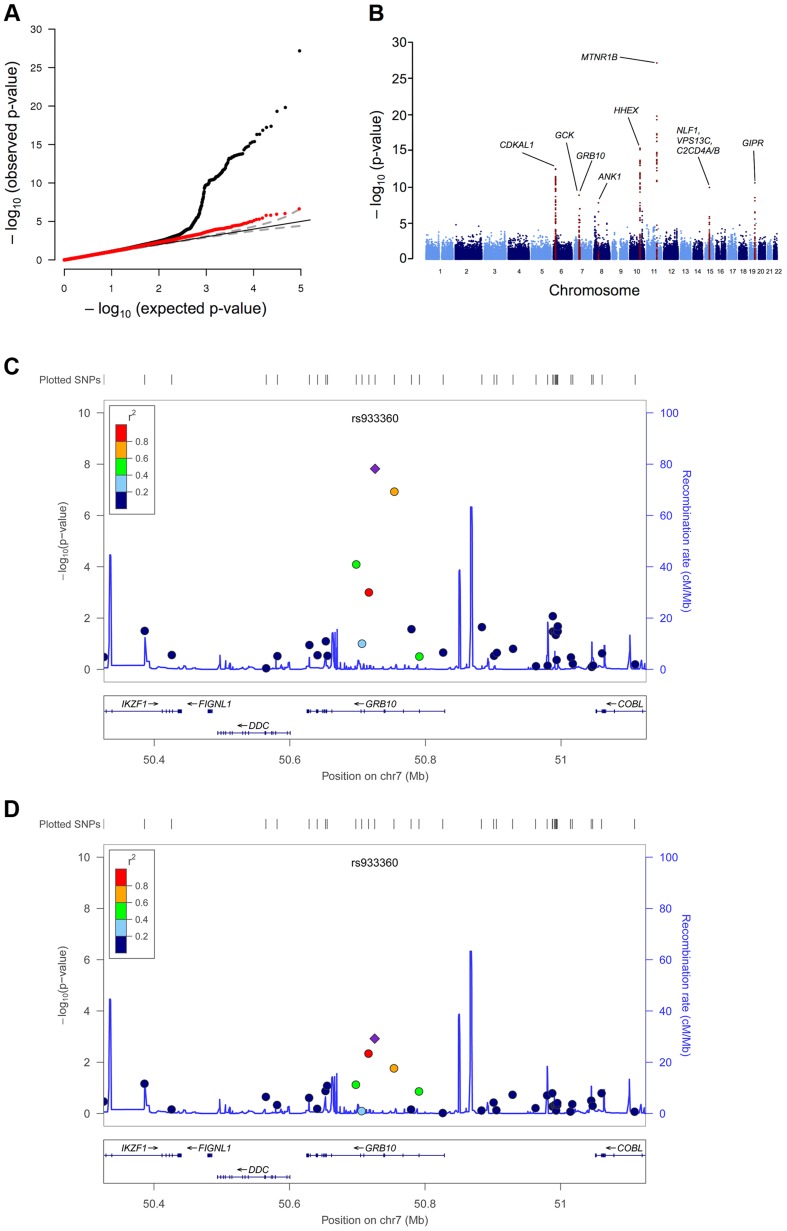
GWAS plots identifying *GRB10* rs933360. Genome-wide quantile-quantile (Q-Q) (A) and Manhattan (B) plots for CIR in the meta-analysis. Regional plots of identified *GRB10* genome-wide significant association for CIR in men (C) and women (D). Directly genotyped and/or imputed SNPs of *GRB10* are plotted with their meta-analysis *p*-values (as −log10 values) as a function of genomic position (NCBI Build 36). In each panel, the strongest associated SNP is represented by a purple diamond (with meta-analysis *p*-value). Estimated recombination rates (taken from HapMap) are plotted to reflect the local linkage disequilibrium structure around the associated SNPs and their correlated proxies (according to a blue to red scale from r^2^ = 0 to 1, based on pairwise r^2^ values from HapMap CEU). Gene annotations were taken from the University of California, Santa Cruz, genome browser, as implemented in LocusZoom [41].

We conducted a meta-analysis for dynamic measures of insulin response to glucose during an OGTT using GWAS, CardioMetabochip and *de novo* genotyping. The combined analysis in up to 26,037 non-diabetic individuals provided genome-wide significant association (*p*<5×10^−8^) with insulin secretion measured as corrected insulin response (CIR) to glucose at 30 min during an OGTT for the locus within the *GRB10* gene (lead SNP rs933360 located in intron 2) (Figure 1A, B), and at 7 previously reported T2D and glycemic trait variants, including *MTNR1B*, *HHEX/IDE/KIF11*, *CDKAL1*, *GIPR/QPCTL*, *C2CD4A (NLF1)*, *GCK* and *ANK1* (Table 1, Table S2A, Figure S1, S2). These associations remained virtually unchanged when CIR was adjusted for insulin sensitivity (disposition index) (Table S2A). In addition, nominal (*p*<0.05) association with reduced insulin response to glucose (CIR) during the OGTT was seen for the risk alleles in 24 out of 65 reported T2D loci [4], as well as for 20 glucose and 7 insulin loci out of 53 reported being associated with these traits [6]. Notably, the risk alleles in 5 of them (*ANKRD55*, *GRB14*, *PPP1R3B*, *IRS1* and *ARAP1* (fasting glucose variant)) were associated with higher insulin response to glucose (*p*<0.05) (Table S2B).

**Table 1:** SNPs associated with primary insulin secretion traits at genome-wide significance levels.

SNP	Nearest gene	N	Alleles (effect/other)	Freq (effect allele)	CIR			AUC_Ins_/AUC_Gluc_		
					Effect	SE	*p*	Effect	SE	*p*
Identified in the present study										
rs933360	*GRB10*	26,037	A/G	0.62	−0.051	0.0086	3.14×10^−9^	−0.043	0.0087	8.13×10^−7^
Previously reported loci										
rs10830963	*MTNR1B*	10,651	G/C	0.69	−0.17	0.016	6.71×10^−28^	−0.057	0.017	0.00062
rs7923866	*HHEX/IDE*	10,418	C/T	0.62	−0.12	0.015	4.16×10^−16^	−0.093	0.015	1.37×10^−9^
rs7756992	*CDKAL1*	10,829	G/A	0.3	−0.11	0.015	3.07×10^−13^	−0.067	0.016	4.16×10^−5^
rs11671664	*GIPR*	9,453	A/G	0.11	−0.17	0.025	2.64×10^−11^	−0.17	0.026	2.63×10^−11^
rs4502156	*C2CD4A (NLF1/VPS13C)*	10,787	T/C	0.52	−0.092	0.014	1.14×10^−10^	0.0035	0.015	0.82
rs3757840	*GCK*	10,322	T/G	0.45	−0.090	0.015	1.34×10^−9^	−0.083	0.016	1.30×10^−7^
rs12549902	*ANK1*	14,834	A/G	0.58	−0.060	0.010	1.01×10^−8^	−0.036	0.011	0.00083

The results are from the meta-analyses of the discovery GWAS, CardioMetabochip and *de novo* genotyping. Results are reported for the directly genotyped and imputed SNPs tested for association with insulin secretion measured as CIR and AUC_Ins_/AUC_Gluc_ (trait abbreviations are listed in the Methods “Phenotype definition” section). Freq denotes the allele frequency of the insulin secretion-reducing allele. N = sample size. Since the index SNP rs933360 (A/G) from the discovery GWAS was not present on the CardioMetabochip platform, a variant (rs6943153 (C/T)) in strong LD with the former (r^2^ = 0.82) was used as a proxy SNP for the meta-analyses.

In line with a previous report [6], we confirmed the association of the index *GRB10* SNP rs933360 with fasting glucose levels (N = 24,608, β = −0.016, *p = *0.007) (Figure S3). However, we did not observe a significant effect of SNP rs2237457, previously associated with glucose concentrations and risk for T2D in an Amish population [13].

Since *GRB10* is differentially expressed when transmitted from mothers and fathers, we explored whether the *GRB10* variant would have sex-specific effects on insulin and glucose levels. The sex-stratified analysis showed a greater insulin-reducing effect of the *GRB10* variant in women (CIR: N = 6,202; β = −0.110±0.019; *p = *1.52×10^−8^) than in men (CIR: N = 15,192; β = −0.038±0.012; *p = *0.0012; sex heterogeneity *p = *0.0016) (Figure 1C, D).

### Complex Pattern of Genetic Inheritance for Variants in the GRB10 Gene: Evidence for Parent-of-Origin Effects

Given the complex parent-of-origin imprinting pattern described for *GRB10*, we next turned to families to explore in detail the inheritance patterns of identified variants and their potential effect on risk of T2D and insulin/glucose levels. Using a cohort of 2,322 parents-offspring trios with 4,182 individuals from Finland and Sweden, we first performed a transmission disequilibrium test, taking into account the parental phenotype being either T2D or not, to increase power (parenTDT) [14]. We also used gene-dropping permutation to control for stratification and the dependence of related individuals [14], or restricting the analysis to independent trios, including only the youngest affected offspring (N = 1,055; 182 with T2D) and oldest unaffected offspring (N = 1,019; 873 unaffected). We observed an increased transmission of the A-allele of rs933360 from parents to diabetic offspring (*p = *0.0063) (Table S3A-I), particularly from diabetic fathers (*p = *0.049) (Table S3A-V), and the G-allele was preferentially transmitted from non-diabetic parents to non-diabetic offspring (*p = *0.026) (Table S3A-II). In accordance with these findings, when simply counting transmission of the A- and G-alleles, we observed an increased transmission of the major A-allele of rs933360 from a diabetic parent to a diabetic offspring (Chi-square *p = *0.017) (Table S3A-V). This effect was even stronger when we relaxed the definition of hyperglycemia to include IFG and IGT in addition to T2D (*p = *0.006) (Table S3A-VI).

We observed a similar pattern of an increased transmission of the T-allele of rs6943153 (LD with rs933360; D′ = 0.99, r^2^ = 0.82) to a hyperglycemic offspring (IFG/IGT/T2D) (*p = *0.0045) (Table S3A-III). This latter association was confirmed using the Family Based Association Test (FBAT) [15] (*p = *0.035), which can accommodate any type of genetic model and family construction. Consistent with previous findings [6], we confirmed the association of the SNP rs6943153 with fasting glucose levels (1,083 nuclear families, *p = *0.02) (Table S3A-VII).

**Figure 2:**
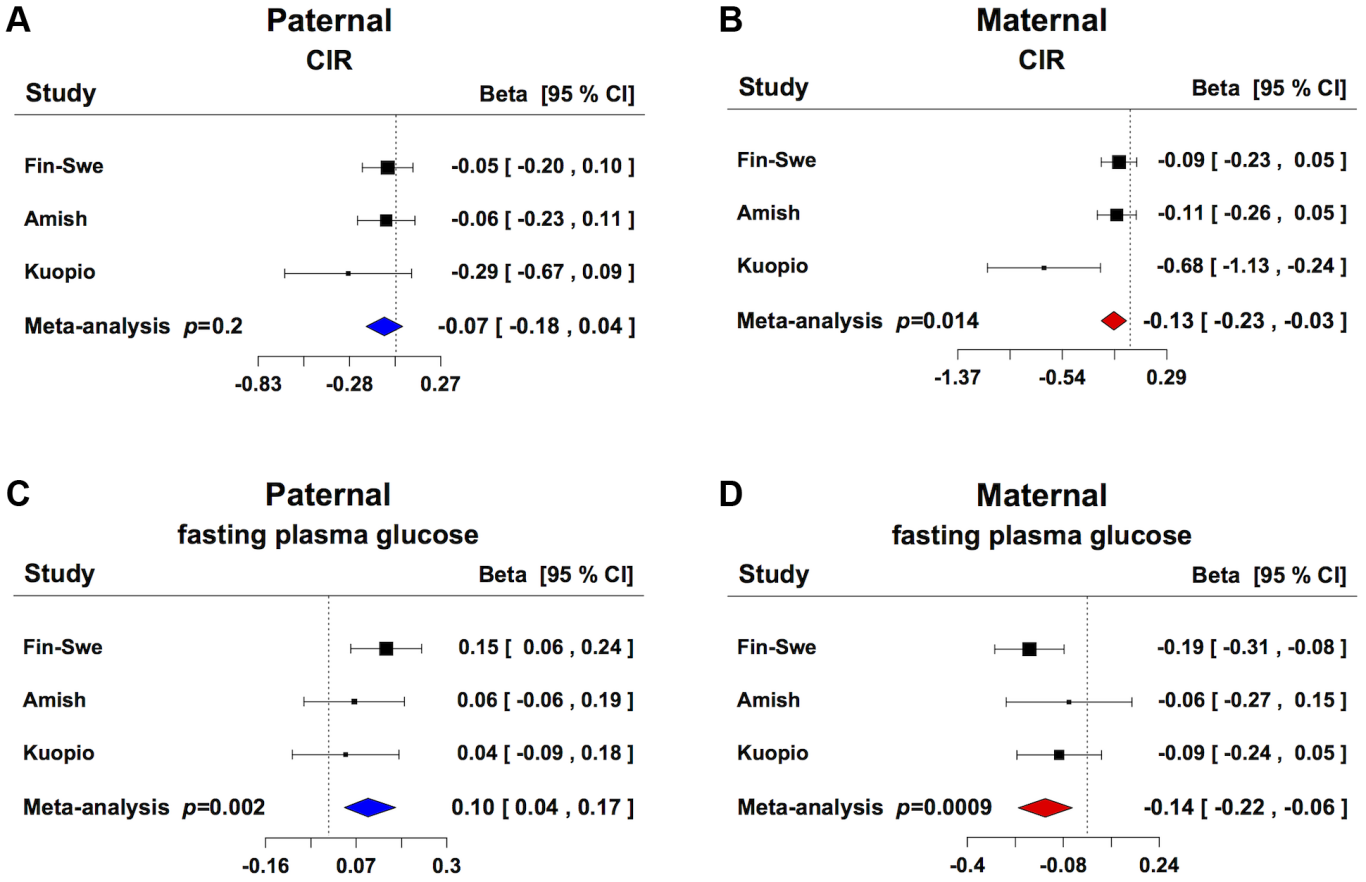
Parent-of-origin effect of *GRB10* rs933360 on insulin secretion and glucose levels. (A) No significant effect for CIR was observed from the paternally transmitted A-allele. (B) Carriers of the maternally transmitted A-allele showed lower CIR compared to the G-allele. (C) Carriers of the paternally transmitted A-allele had elevated fasting plasma glucose levels, whereas (D) the maternally transmitted A-allele was associated with lower fasting plasma glucose levels. Fin-Swe = Trios from Finland and Sweden, Amish = Amish Family Diabetes Study, Kuopio = Kuopio Offspring Study.

To explore whether *GRB10* rs933360 would show a stronger effect on insulin secretion when inherited from either parent, we examined its effect on GSIS in 3,117 non-diabetic individuals from parents-offspring trios from Finland and Sweden [16] and USA [13]. In these families, the maternally transmitted A-allele of rs933360 was associated with reduced GSIS (CIR β = −0.127, *p = *0.014; Ins30adjBMI β = −0.125, *p = *0.005; Ins30 β = −0.112, *p* = 0.014; AUC_Ins_ β = −0.095, *p = *0.016; AUC_Ins_/AUC_Gluc_ β = 0.107, *p = *0.005) (Figure 2A, B, Table S3B). No significant effect was observed for the paternally transmitted A-allele on GSIS. Surprisingly, the maternally transmitted A-allele was associated with reduced rather than elevated fasting glucose levels (β = −0.139, *p = *0.0009). In contrast, the paternally transmitted A-allele was associated with elevated glucose levels (β = 0.102, *p = *0.002) (Figure 2C, D, Table S3B). Thereby, the A-allele of rs933360 exerted virtually opposite effects on glucose metabolism if transmitted from the father than the mother. It is very likely that the association with risk or protection from T2D would be missed or diluted in any traditional association study, which does not take the transmission pattern into account.

In support of this, we did not observe any association between the SNP rs933360 and T2D in 16,715 non-diabetic individuals, of whom 2,637 developed T2D during a mean 25-year follow-up period (Table S4) [17]. Also, in the recent DIAGRAM+ meta-analysis, none of the evaluated *GRB10* SNPs were associated with T2D [4].

A potential explanation for the paradoxical reduction in glucose levels despite reduced insulin secretion could be that the variant also enhances insulin sensitivity or reduces glucagon levels. In fact, the maternally transmitted A-allele was associated with enhanced, whereas the paternally transmitted A-allele was associated with decreased insulin sensitivity as measured by ISI during the OGTT (*p*<0.05 for difference between parental alleles). Although we could not observe any significant effect of rs933360 on fasting or 2 hr glucagon levels in a Finnish cohort with glucagon data available (Table S5B), we identified several *GRB10* SNPs from the same haplotype block which were in weak LD with rs933360 and nominally (*p*<0.05) associated with fasting and 2 hr glucagon levels in the DGI GWAS (Table S6). Unfortunately, there was no glucagon data available for the trios.

### Effects of GRB10 rs933360 on Gene Expression

**Figure 3:**
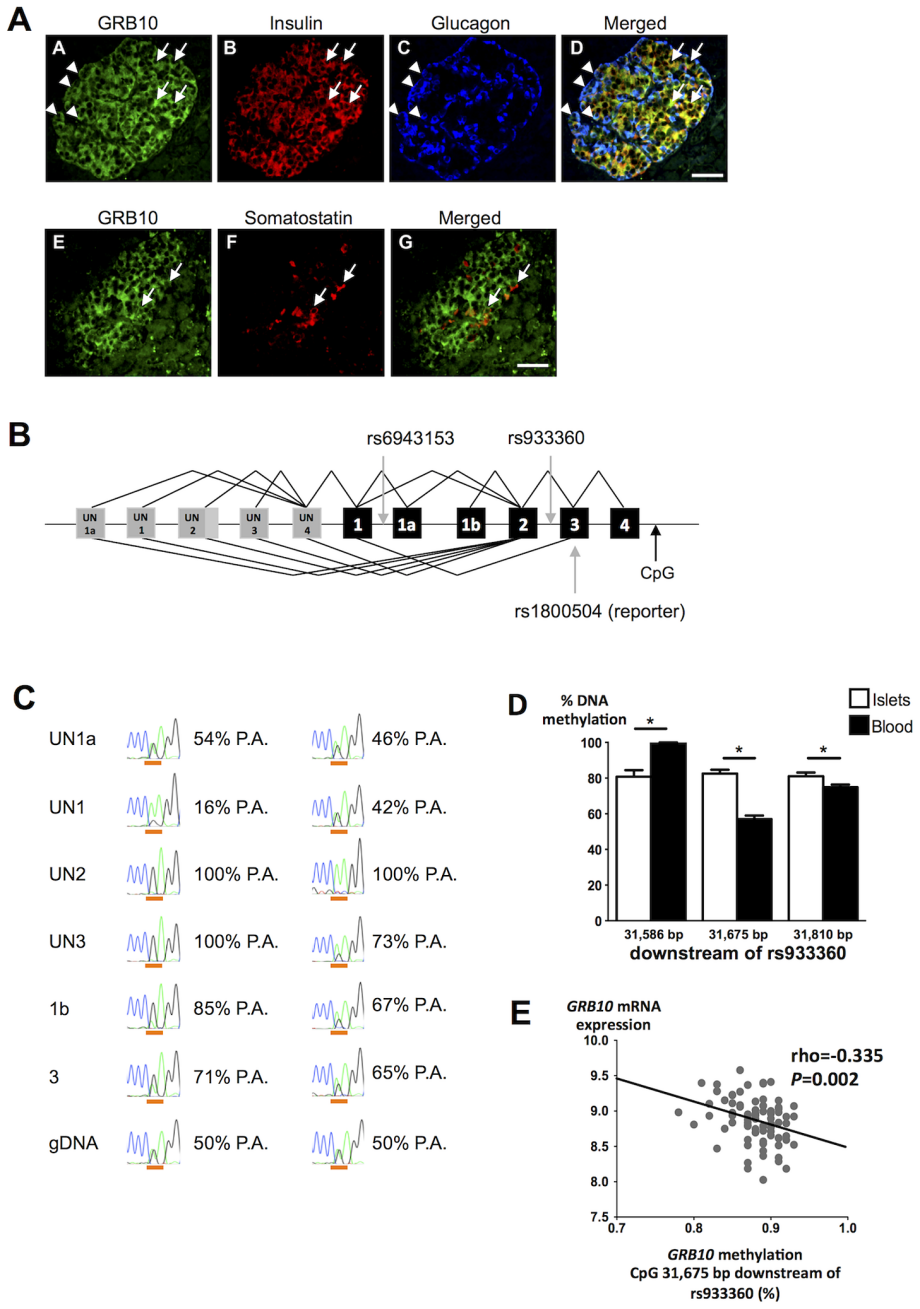
*GRB10* expression in human islets. (A) Immunostainings demonstrating that *GRB10* (green, panel A, E) is abundantly expressed in β- (panel B, D), α- (panel C, D) and δ-cells (panel F, G) in human pancreatic islets. Arrows indicate co-localization with insulin (panel D) and somatostatin (panel G). Arrowheads indicate co-localization with glucagon (panel D). Scale bar = 50 µm. (B) Schematic representation of the *GRB10* gene and SNPs investigated in the present study. Grey boxes = untranslated exons. Black boxes = translated exons. (C) Examples of RT-PCR on islet cDNA (top six rows) and PCR on genomic DNA (gDNA, bottom row) from two individuals heterozygous for the reporter SNP rs1800504. The first column states the forward primer location of each PCR and a forward primer in exon 3 captures all transcripts. The peaks show the Sanger sequencing trace across rs1800504, which is underlined (A: green trace, G: black trace). Percentages indicate the contribution from the paternal allele (P.A.) (G-allele in the first case, A-allele in the second case). The paternal genotype is identified assuming complete maternal imprinting of the UN2 promoter, in line with previous findings [12]. A sequence of heterozygous genomic DNA (gDNA) is shown on the bottom for comparison (50%-50%). (D) To study if DNA methylation of *GRB10* is tissue-specific, the degree of methylation was analyzed at 3 CpG sites located ∼31.7 kb downstream of rs933360 in both human pancreatic islets of 98 donors and PBL from 6 trios using EpiTYPER. The exact position of each analyzed CpG site in relation to rs933360 is given in the figure. Data are presented as mean ± SEM. * *p*<0.05 for difference in methylation between human islets and PBL. (E) The *GRB10* mRNA levels correlated negatively with the degree of methylation at the CpG site located 31,675 bp downstream of *GRB10* rs933360.

GRB10 protein was detected in human α-, β- and δ-cells by immunofluorescence (Figure 3A). We observed strong expression of *GRB10* mRNA in total human islets, with no significant difference between islets from normoglycemic and hyperglycemic individuals (Figure S4A), or between carriers of different *GRB10* genotypes (Figure S4B).

While we did not observe any correlation between the amount of *GRB10* and *INS* (insulin) mRNA, nor between *GRB10* mRNA and *in vitro* GSIS, there was an inverse correlation between *GRB10* and *GCG* (glucagon) mRNA in human pancreatic islets (all donors: rho = −0.267, *p = *0.017; normoglycemic: rho = −0.228, *p* = 0.10; hyperglycemic: rho = −0.651, *p = *0.00003), suggesting that higher *GRB10* expression is associated with lower glucagon (Figure S4C).

Although there was no effect of rs933360 on total *GRB10* mRNA expression in human islets, we cannot exclude that the variant could influence splicing or methylation, especially as 3 different transcriptional start sites (UN1, UN1a and UN2) and tissue-specific expression have been described for the *GRB10* gene (Figure 3b, S5) [12].

We tested for allelic imbalance, i.e. deviation from the expected equal expression of both alleles. For this purpose, we used the SNP rs1800504 (A→G) located in exon 3 as a reporter SNP, as it is the nearest coding variant located 16 kb from the index SNP rs933360 (D′ = 1, r^2^ = 0.5). This reporter SNP indicated a clear allelic imbalance with A- to G-allele ratios ranging from 35% to 75% in pancreatic islets (Table S7). We therefore examined whether the observed allelic imbalance could be related to specific transcripts arising from different promoters. Transcripts containing exon UN2 were monoallelically expressed from either the A- or G-allele, indicating imprinting of the promoter giving rise to the UN2 transcripts (p_UN2_) from one parent. Until now, paternally expressed (i.e. maternally imprinted) UN2 transcripts have only been observed in the brain [12]. This differential imprinting was recurring in all tissues analyzed (Figure S6). Our findings extend this expression pattern to human islets. In contrast, transcripts containing exon UN1 showed great variation (from 50% to 80%), but were mainly expressed from the other allele than those containing exon UN2 (Figure 3B, C), in line with the maternally expressed/paternally imprinted transcripts observed in placental trophoblasts [12]. It can be hypothesized that these SNPs might regulate usage of alternative promoters and thereby influence the expression of *GRB10*. The opposite effects of maternally and paternally inherited rs933360 allele could then be attributed to different effects of rs933360 on the promoters p_UN2_ and p_UN1_, e.g. especially if promoter preferences differ strongly between α- and β-cells.

Although allelic imbalance is an attractive model to explain differences in expression of different *GRB10* transcripts, as well as the observed differences in effects of the A-allele on risk of diabetes in the offspring when transmitted from father or from mother, the above data can only point at this possibility, as we did not have enough human islets for this kind of analysis. We therefore also tested for allelic imbalance using an alternative method, i.e. by comparing data from exome and RNA sequencing. We found that another coding variant, SNP rs11555134, in the *GRB10* gene was associated with allelic imbalance in 8 human pancreatic islet samples (*p*<0.05, Fisher's exact test).

Since *GRB10* is imprinted and methylated in humans and rodents in a tissue-specific fashion [12], [18], [19], we studied whether *GRB10* would be methylated in human islets or in DNA from human peripheral blood lymphocytes (PBL) and whether the degree of the DNA methylation would correlate with gene expression in human islets. We found tissue-specific differences in DNA methylation of *GRB10* in human islets compared to PBL and the degree of methylation in the region analyzed with the Sequenom MassARRAY EpiTYPER ranged from 56.9% to 99.8% (Figure 3D). Although we did not observe any significant effect of rs933360 on the degree of DNA methylation in human islets (Figure S7A), there was a nominal association between rs933360 and DNA methylation in PBL in the region analyzed using EpiTYPER (*p* = 0.07) (Figure S7B). Moreover, we observed an inverse correlation between DNA methylation at a CpG site located 31.7 kb downstream of rs933360 and *GRB10* mRNA expression (N = 81, rho = −0.335, *p* = 0.002) (Figure 3E) in human islets, particularly in islets from diabetic donors (N = 24, rho = −0.656, *p* = 0.001), and at a CpG site 8,196 bp downstream from rs933360 (N = 66, rho = −0.23, *p* = 0.047) (Figure S7C), suggesting that decreased methylation in this region is associated with increased *GRB10* mRNA.

### Expression of GRB10 in Human Muscle and Adipose Tissue

Given that we observed differences in insulin sensitivity when the risk A-allele of SNP rs933360 was inherited from the mother compared to the father, and that GRB10 is an inhibitor of insulin signaling, we also explored whether the SNP would influence expression of the *GRB10* gene in human skeletal muscle and adipose tissue [20], [21]. We observed that carriers of the A-allele had decreased *GRB10* mRNA level in muscle (N = 38, β = −0.405, *p* = 0.013) and adipose tissue (N = 1,375, β = −0.038, *p* = 0.005).

### Effects of GRB10 on Islet Function

**Figure 4:**
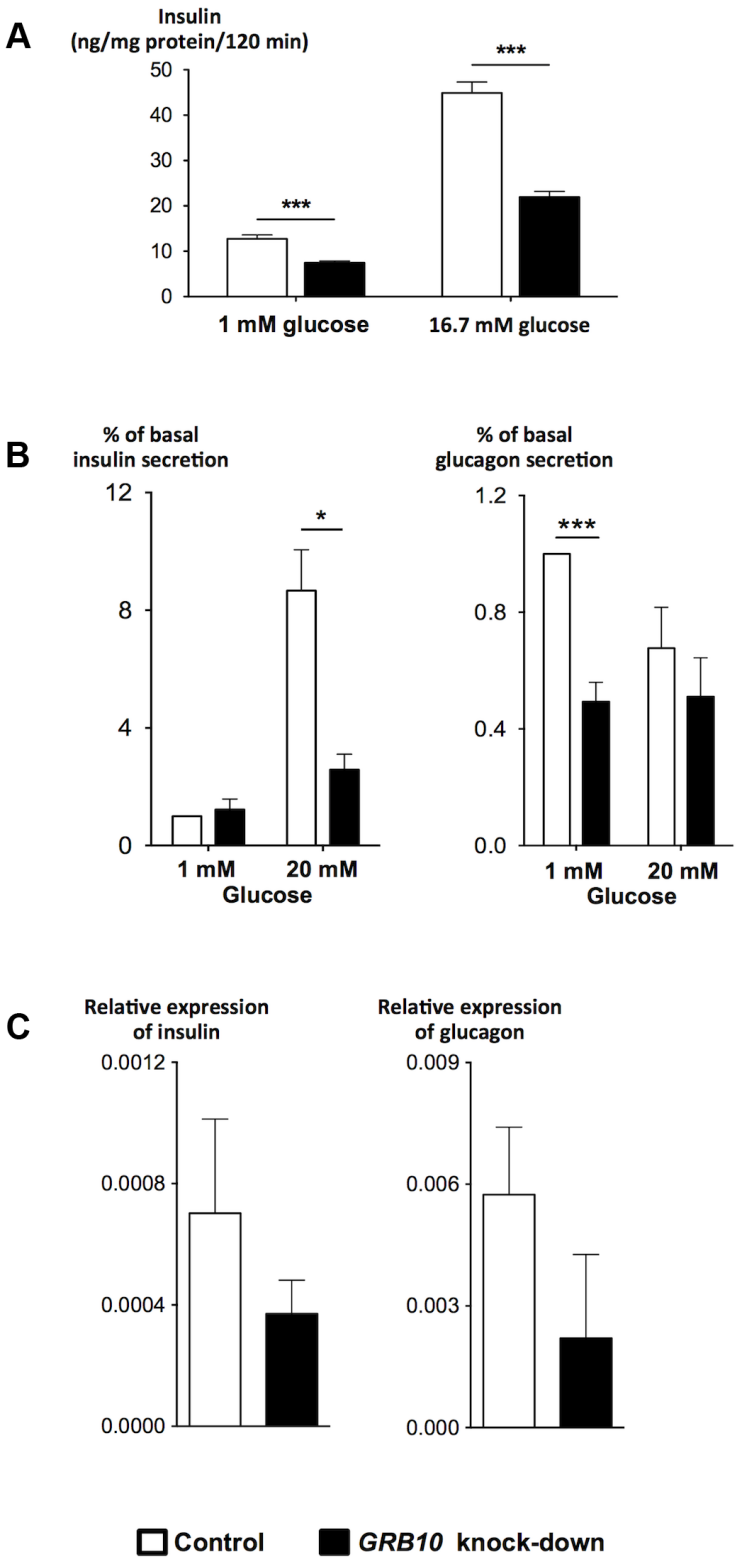
Effects of disrupted *GRB10* through knock-down on islet function. (A) Disrupted *GRB10* in INS-1 rat β-cells markedly reduced glucose-stimulated insulin secretion. (B) *GRB10* knock-down showed reduced glucose-stimulated insulin secretion at 20 mM glucose and glucagon secretion at 1 mM glucose in human pancreatic islets (N_insulin_ = 7, N_glucagon_ = 6 donors of human pancreatic islets; 3–6 measurements in each experiment for each donor). (C) *GRB10* knock-down resulted in a reduction of insulin and glucagon mRNA expression (N = 3 donors of human pancreatic islets; 3 measurements in each experiment for each donor). * *p*<0.05; ** *p*<0.01, *** *p*<0.001. Error bars denote SEM.

To gain insight into the mechanisms by which *GRB10* influences pancreatic β- and α-cell function, we disrupted *Grb10* expression in rat insulinoma INS-1 cells by siRNA and in human islets by shRNA achieved by lentiviral transfection. There was a clear reduction in GSIS after siRNA-disruption of Grb10 in the INS-1 cell line lacking glucagon (Figure 4A). In human pancreatic islets, decreased *GRB10* expression resulted in a reduction of both insulin and glucagon secretion and expression (Figure 4B, C). In addition, *GRB10* knock-down was also associated with a decrease in forskolin- and K^+^-stimulated glucagon secretion (Figure S8A).

Grb10 has been reported to both increase [22] and decrease [23] apoptosis in islets, in addition to its effects on insulin signaling [22]. Disruption of *GRB10* was associated with a significant reduction in the number of viable human pancreatic islets, as assessed by an MTS technique, supporting recent data in mice using shRNA to disrupt *Grb10* in pancreatic islets [22] (Figure S8C).

## Discussion

The present study provides mechanistic insights into the role of *GRB10* in the regulation of islet function and glucose metabolism in man. First, the A-allele of the *GRB10* rs933360 variant was associated with different effects on insulin secretion, sensitivity and glucose concentrations, if transmitted from fathers or mothers. Second, disruption of *GRB10* in human islets resulted in a reduction of both insulin and glucagon secretion, the latter of which can provide an additional explanation for the reduction in glucose levels despite reduced insulin levels. Third, metabolic effects of *GRB10* on glucose homeostasis involved tissue-specific methylation and parental imprinting.

GRB10 is an adaptor protein, which interacts with a number of receptor tyrosine kinases and signaling molecules and serves as a down-regulator of insulin receptor activity [24]. GRB10 participates in phosphorylation and activation of the mTORC1 protein, which is a central regulator of cellular metabolism, growth and survival [25]. Studies in mice have shown an abundant expression of *Grb10* in brain, fat, muscle and heart, and with the highest expression in pancreas [26].

An interesting observation in the present study was that the common variant rs933360 in the *GRB10* gene was associated with reduced GSIS, enhanced insulin sensitivity and reduced glucose levels when inherited from the mother. On the contrary, the paternally transmitted allele was associated with elevated glucose level and increased risk for T2D. These findings might partially explain the modest or lack of effects seen in several studies for variants in the *GRB10* gene on T2D risk [27]. The effect on insulin sensitivity is indirectly supported by the view that carriers of the A-allele showed decreased expression of *GRB10* in skeletal muscle and adipose tissue. Another potential explanation could be the effect of *GRB10* on glucagon secretion. Unfortunately, due to lack of samples for glucagon measurements, we were not able to test this directly in the trios.

To further explore whether *GRB10* influences glucagon secretion/expression and thereby mechanisms by which *GRB10* might influence glucose metabolism, we disrupted *GBR10* in human pancreatic islets. While *Grb10* knock-down in the rat insulinoma cell line INS-1 lacking glucagon resulted in marked reduction of insulin, *GRB10* knock-down in human pancreatic islets resulted in reduction in both insulin and glucagon secretion and expression. The effect on glucagon seen in human islets is in line with data from mice, showing reduced glucagon levels after *Grb10* disruption using shRNA [22].

Since rs933360 is located in intron 2 of the *GRB10* gene, one could envision an effect on gene expression. This could be a possible mechanism, as monoallelic and isoform-specific expression of *GRB10* has been reported in fetal brain, skeletal muscle and in the placental trophoblasts [11], [12]. Unfortunately, we could not observe any effect of the SNP on expression of the *GRB10* transcript in islets, nor could we examine parent-of-origin effects in the islets from human cadaver donors, as we lacked information on the parents. However, using two different strategies we could demonstrate allelic imbalance for SNPs in the *GRB10* gene, leaving this possibility open. In line with earlier findings, we also identified three transcripts arising from alternative *GBR10* promoters, with the paternally expressed UN2 isoform being imprinted from one parent in islets. However, there is sufficient amount of the maternally expressed UN1a isoform in islets to allow an effect of the maternally transmitted allele, which could contribute to the stronger effect of the maternal allele as described above.

Genetic imprinting is usually a consequence of increased DNA methylation. In line with previous reports [11], [12], we observed tissue-specific methylation of *GRB10*, showing highest degree of methylation in human pancreatic islets, which was associated with decreased expression of *GRB10.* Although this relationship was not influenced by the SNP in human pancreatic islets, there was a tendency for an effect of the SNP on degree of methylation in PBL.

Our study has some limitations. Despite large sample size for measurements of insulin secretion, we did neither have samples with measurements of glucagon in the trios, nor did we have parental information for islet donors. Also, tissue limitations prevented us from directly exploring methylation patterns and imprinting in trios. Finally, although this is the largest meta-analysis for insulin secretion to date, we discovered only one novel locus influencing GSIS. While this could be a consequence of limited power, another plausible explanation is that many homeostatic mechanisms, as well as parent-of-origin specific effects, would dilute and neutralize such effects.

In conclusion, our data demonstrate a complex genetic regulation of *GRB10* function in human islets with different effects of paternally and maternally transmitted alleles. Together, these findings suggest that tissue-specific methylation and possibly imprinting of *GRB10* can influence glucose metabolism and contribute to T2D pathogenesis. The data also emphasize the need in genetic studies to consider whether disease risk alleles are inherited from the mother or the father.

## Methods

### Ethics Statement

All studies were approved by local research ethics committees, and all participants gave informed consent. All procedures in human islets were approved by the ethics committees at the Uppsala and Lund Universities and informed consent obtained by appropriate measures from donors or their relatives.

### Meta-Analyses of Genome-Wide Association and CardioMetabochip Studies

Association analyses of insulin secretion and action traits were performed within 11 cohorts participating in the Meta-Analysis of Glucose- and Insulin-related traits Consortium (MAGIC) in a total of up to 10,831 individuals. In the discovery stage 1, we performed a meta-analysis of 6 GWASs (Diabetes Genetics Initiative (DGI), Amish Family Diabetes Study, Sorbs, Helsinki Birth Cohort Study (HBCS), French Obese Adults, and Relationship between Insulin Sensitivity and Cardiovascular disease Study (RISC)) for glucose-stimulated insulin secretion (GSIS) during an oral glucose-tolerance test (OGTT) at 3 time points (fasting, 30 min, 120 min) for primary traits measured as (1) insulin response to glucose after the first 30 min estimated as corrected insulin response (CIR), and (2) overall insulin response to glucose estimated as area under the curve (AUC) for insulin over a total AUC for glucose (AUC_Ins/_AUC_Gluc_) in up to 5,318 non-diabetic individuals (Table S1A).

As none of the traits gave genome-wide significant association, we selected the top 50 independent signals from both primary and secondary traits (see “Phenotype Definition” below) after LD pruning (r^2^<0.2). Signals prioritized for replication were ranked by the number of associations observed at primary traits and/or secondary traits, association p-value and number of times the signal was observed across the traits (more than 2). We selected 14 SNPs for replication genotyping and follow-up analyses, out of which 3 loci were based on biological relevance: *GRB10* [13] (rs933360, discovery *p*-value (CIR) = 5.09×10^−6^), *UCN3* (rs11253130, discovery *p*-value (Ins30adjBMI) = 9.46×10^−7^) and *INADL* (rs2476186, discovery *p*-value (AUC_Ins_/AUC_Gluc_) = 1.88×10^−6^). Replication stage 2A *de novo* genotyping was undertaken in five population-based studies (Botnia-PPP, ULSAM, METSIM, BPS and Haguenau; only GWAS index SNP rs933360 in the latter three; max N = 15,273) (Table S1A, S2A). Replication stage 2B *in silico* was undertaken using an iSelect CardioMetabochip array (CM) (Illumina, San Diego, CA, USA) to genotype data in 5 independent population-based studies (Botnia-PPP, ULSAM, Ely, DR's Extra and METSIM) including up to 5,513 individuals (Table S1A).

The GWAS/CM (stage 1 and stage 2B) data including 93,896 SNPs were pooled together with the *de novo* genotyping results from stage 2A for non-overlapping individuals. In this meta-analysis, we defined all independent (r^2^<0.2) genome-wide significant (*p*-value<5×10^−8^) association signals for insulin secretion traits at 8 genomic loci (Table 1).

### Phenotype Definitions

The primary insulin secretion and action indices were: (i) Corrected Insulin Response (CIR) = (100× insulin at 30 min)/(glucose at 30 min×(glucose at 30 min–3.89)), and (ii) ratio of the area under the curve (AUC) for AUC insulin/AUC glucose (AUC_Ins_/AUC_Gluc_, mU/mmol) calculated using the trapezium rule [28]. Insulin sensitivity index (ISI) = 10,000/√ (fasting plasma glucose (mg/dl)×fasting insulin×mean glucose during OGTT (mg/dl)×mean insulin during OGTT). Secondary insulin secretion and action indices during OGTT were: (i) disposition index (DI) = CIR×ISI; (ii) insulin at 30 min (Ins30); (iii) incremental insulin at 30 min (Increm30) = insulin 30 min – fasting insulin; (iv) insulin response to glucose during the first 30 min adjusted for BMI (Ins30adjBMI) = insulin at 30 min/(glucose at 30 min×BMI); (v) area under the curve (AUC) of insulin levels during OGTT (AUC_Ins_, mU*min/l). Individuals with missing data on any of the three time points included in the AUC calculation were excluded.

### Statistical Analysis

Linear regression models were used for association of phenotypes (z-score residuals of insulin secretion and action traits) with genotypes coded additively. Discovery (stage 1) GWAS analyses were carried out using a statistical tool that was able to account for genotype uncertainty, SNPTEST [29], or by using allele dosages in the linear regression model in MACH2QTL [30], [31], probABEL [32], corrected for residual inflation of the test statistics using the genomic control method [33]. The meta-analyses of effect sizes were performed with the fixed-effect inverse-variance method using GWAMA [34]. The GC correction was applied only once to cohort-specific results before including them into the meta-analyses. Sex-differentiated analyses were performed using GWAMA, with an assumed heterogeneity *p*-value of <0.05. Effect sizes for glucose levels were estimated using a fixed-effect model using the metaphor package for R version 2.14.2 (http://www.r-project.org/).

### Parent-of-Origin Effect Analysis on Insulin Secretion and Glucose Levels

The Trios from Finland and Sweden, Amish Family Diabetes Study and Kuopio Offspring Study (Table S1A, S3A, B) consisted of a father, a mother and an offspring. Genotype phase was determined using Merlin and then analyzed using Solar, which uses the kinship matrix to account for family. Meta-analysis on insulin secretion and action and glucose levels during OGTT (as earlier described) was performed using a fixed-effect model. Analyses were performed on IBM SPSS Statistics 20.0 (IBM Corp., Chicago, IL, USA), R version 2.14.2 with the metaphor package (http://www.r-project.org/) and MMAP (MMAP: mixed models analysis for pedigrees and populations, http://edn.som.umaryland.edu/mmap/index.php).

The Transmission Disequilibrium Test (TDT) used to compare frequencies of transmission of the two alleles from heterozygote parents to an affected offspring was performed using PLINK (http://pngu.mgh.harvard.edu/purcell/plink/) [35] (Table S3A). The deviations from Mendelian transmissions were assessed and the power of the test was enhanced by incorporating information from phenotypically discordant parents (ParenTDT) [14]. To confirm the association, another independent test was performed, which can accommodate any type of genetic model and family construction, i.e. the Family Based Association Test (FBAT) [15]. Quantitative traits related to glucose metabolism and insulin secretion were assessed in non-diabetic individuals using qTDT and parent-of-origin effect tests. IBD estimates were calculated using Merlin. Permutations were performed using QFAM (PLINK). OGTT values were natural log-transformed and adjusted for BMI.

### Association of GRB10 rs933360 with Glucagon Levels

The Botnia Prevalence, Prediction and Prevention of diabetes (Botnia-PPP) study is a population-based study from the Botnia region of Western Finland and has previously been described [36]. For this study, we selected 4,641 non-diabetic individuals above the age of 18. Linear regression analysis assuming an additive genetic risk model was performed to evaluate genotype-phenotype association. Hyperglycemic individuals were identified based on previous diagnosis, fasting plasma glucose levels of 5.5–6.9 mmol/l and 2 hr plasma glucose levels of 7.8–11.1 mmol/l (Table S5).

### Association of GRB10 rs933360 with GRB10 mRNA in Human Muscle

A subgroup of 203 men with IGT at screening visit selected from the MPP study participated 20 years later in more extensive metabolic studies, including a new OGTT, a euglycemic-hyperinsulinemic clamp combined with indirect calorimetry and infusion of [3-3H]glucose [37], [38]. The men were similar in age, but had varying degrees of glucose tolerance; 69 were in the normal range, 52 had IFG and/or IGT, and 82 had T2D. T2D patients were either treated with diet alone (42%) or with oral hypoglycemic agents, which were withheld the day before the test. Microarray expression data were analyzed as previously described [20].

### Association of GRB10 rs933360 with Future Risk for T2D

The Malmö Preventive Project (MPP) is a large population-based prospective study from the city of Malmö, Sweden, and has previously been described [17]. For this study, we selected 16,715 non-diabetic subjects, of whom 2,637 developed T2D during a 24.1 year mean follow-up period. The odds ratio for risk of developing T2D was calculated using logistic regression analysis assuming an additive genetic risk model. The analysis was adjusted for age, sex, BMI, participation period and an interaction term (participation period x sex) [10]. IBM SPSS Statistics 20.0 (IBM Corp.) was used for the statistical analysis.

### Human Pancreatic Islets

Islets from cadaver donors were provided by the Nordic Islet Transplantation Program (www.nordicislets.org) by the courtesy of Prof. Olle Korsgren, Uppsala University, Sweden. The microarray experiments (Human Gene 1.0 ST whole transcript) were performed on islets isolated from 81 normoglycemic (mean±SEM; age 56.3±1.3 yrs, BMI 25.7±0.4 kg/m^2^, HbA1c 5.5±0.04%) and 46 hyperglycemic (age 60.3±1.2 yrs, BMI 27.8±0.6 kg/m^2^, HbA1c 6.6±0.1%) islet donors. RNA products were fragmented and hybridized to the GeneChip Human HG U 133A Array (Affymetrix, Santa Clara, CA, USA) [39]. Statistical analyses of expression data were performed using two-tailed Spearman's T-test.

### Immunocytochemistry

Immunocytochemistry with antibodies for GRB10 (K20)^18^ (code sc-1026, Santa Cruz Biotech. Inc., CA, USA), insulin, glucagon and somatostatin was performed on human pancreatic sections as previously described [40].

### RNA Sequencing of Human Islets

RNA from 48 human pancreatic islets donors (24 with HbA1c <5.5% and 24 donors with HbA1c >6.0%) was isolated and purified using miRNeasy kit (Qiagen, Hilden, Germany). As quality thresholds for RNA samples, we demanded RIN values >8, 28S/18S ratio >1.5 and the absorbance ratios 260/280 >1.8 and 260/230 >1. Sample preparation for sequencing reactions was performed using TrueSeq sample prep kit (Illumina). Fragmentation was performed using inbuilt fragmentation in the sample prep kit to obtain fragments of approximately 300 bp in length. Sequencing was performed on the Illumina HiSeq 2000 platform. Obtained sequenced reads were transformed into .qseq files using the Illumina pipeline. Alignment of the reads was performed using the TopHat short read aligner (tophat.cbcb.umd.edu). Cufflinks (cufflinks.cbcb.umd.edu) was used for splice variant calling.

### Identification of GRB10 Splice Variants and Measurement of Allelic Imbalance

Analytical RT-PCRs were performed on cDNA from human islet, visceral fat, subcutaneous fat, liver and muscle in 15 µl reactions using 7.5 µl AmpliTaq Gold PCR Master Mix (Applied Biosystems, Foster City, CA, USA) supplemented with 0.75 µl DMSO and 1.5 µmol/l of forward and reverse primers. The following primers were used in various combinations: UN1fw: CAAACGCCTGCCTGACGACTG, UN1Afw: GCCCGGGACAGTCTTGAGC, UN2fw: GGCGCACACGCAGCGAC, UN3fw: ACCACCTACATCAGAGCTGACTGCC, 1bfw: CCTGGGCTACCCTCTGCTTC, 3fw: GCCTGTACTCGGCCTGCAGC, 9fw: GCCCCTACAGACCACGGGCT, 11fw: GCTGTCCCCGTTCTCGACGC, 3rv: ATGTGCACAGGCTGGGAGCG, 7rv: CTGGCTGTCATGTCTGCT, 11rv: CTGCTGAGGGATTCGGT, 16rv: GGATGCAGTGGTGCTTGA, the names referring to the target exon. PCR reactions were carried out with 53°C annealing temperature and over 50 cycles. Products were analyzed on 2% agarose. Prior to sequencing, 2.5 µl PCR product was treated with 0.5 µl ExoSAP-IT (USB, Cleveland, OH, USA) at 37°C for 30 min followed by deactivation at 80°C for 15 min. Subsequently, 1 µl was sequenced in both directions using BigDye 3.1 according to the manufacturer's protocol (Applied Biosystems). The sequence reactions were purified and analyzed by GATC Biotech AG (Konstanz, Germany).

Allelic imbalance measurements were performed by RT-PCR using the reverse primer 3rv and either of the forward primers UN1afw, UN1fw, UN2fw, UN3fw, 1bfw or 3fw in samples heterozygous for the common SNP rs1800504 (*GRB10* exon3). The individual contribution from each allele was measured at the position of rs1800504 using Sanger sequence traces and the software Mutation Surveyor (Soft Genetics, PA, USA). Allelic imbalance in *GRB10* was also detected by a different method: After extensive quality and coverage filtering, we did a Fisher exact test for comparing the ratio of reference/alternative alleles in the exome sequencing vs. RNA-seq for each sample. Exome sequencing was performed using the Illumina exome sequencing protocols (TruSeq DNA sample preparation Kit v2).

### Methylation Studies

Sequenom's MassARRAY EpiTYPER protocol was applied to measure DNA methylation (Sequenom, San Diego, CA, USA) in human islets of 96 donors and peripheral blood lymphocytes (PBL) of 6 diabetic offspring trios (18 individuals). EpiDesigner was used for assay design at *GRB10* and the primer sequences were the following; forward: aggaagagagGGGAAAGGGTGTTAAATTGTTTATG, reverse: cagtaatacgactcactatagggagaaggctTTTTAAACCCCTCAAATTCAAAAAT. 500 ng genomic DNA was bisulfite-treated with the EZ DNA Methylation kit (Zymo Research, Orange, CA, USA). The spectra were analyzed and the methylation ratios were obtained by the EpiTYPER software v.1.0.1. Global methylation analyses were performed on DNA extracted from PBL on the Illumina Infinium 450 Bead Chip and the chips were scanned on an Illumina iScan as per protocol (Illumina). 1 µg of DNA was bisulfite-treated according to protocol (EZ DNA Methylation Kit, Zymo research). For analysis, the Genome Studio Methylation Module of the Genome studio Genome Browser was used, which facilitates integration of the SNP and CpG location data (NCBI build 37). Methylation status was assessed after normalization to internal controls and background subtraction and expressed as β. The β values for the CpG sites were mapped to the gene and plotted to give an overview of methylation status for the region of interest (Figure S7D).

### Disruption of GRB10 for Insulin and Glucagon Secretion Analyses

#### shRNA-mediated knock-down of GRB10 in human pancreatic islets

Specific silencing of endogenous hGRB10 was achieved using a lentiviral-based shRNA-silencing technique (Santa Cruz Biotech. Inc.). Isolated human islets were incubated at 2.8 mmol/l glucose + Polybree for 90 min. Thereafter, the medium was removed and the islets were washed before culture medium + lentiviral particles containing *GRB10* shRNA (5 µl/ml) was added, and the islets were cultured for 36 h at 5 mmol/l glucose. For comparison, a scramble (lentiviral particles without targeting any specific region) served as control. Insulin and glucagon were measured using a radioimmunoassay kit (Electrobox, Stockhom, Euro-Diagnostica, Malmö, Sweden).

#### siRNA-mediated knock-down of Grb10 in INS-1 832/13 cells

For *Grb10* small interfering RNA (siRNA) experiments, 20–25-nucleotide stealth-prevalidated siRNA duplex designed for rat *Grb10* (Santa Cruz Biotech. Inc.) was used. INS-1 832/13 cells were seeded in 24-well plates at a density of ∼5×105 cells in culture media without antibiotics and transfected with DharmaFECT 1 (Dharmacon, Lafayette, CO, USA) according to the manufacturer's instructions.

### Assessment of β-cell Viability

Human pancreatic β-cell viability assay was performed using a CellTiter 96 AQueous One Solution Cell Proliferation Assay Reagent (Promega, Stockholm, Sweden) according to the manufacturer's instructions. The actual performance is based on the spectrophotometric detection of a colored formazan product converted from a 3-(4,5-dimethylthiazol-2-yl)-5-(3-carboxymethoxyphenyl)-2-(4-sulfophenyl)-2H-tetrazolium (MTS) compound by NADPH or NADH via metabolically active cells.

## Supporting Information

Figure S1 Regional plots for the top 8 hits reaching genome-wide significance level. Association with insulin secretion measured as corrected insulin response (CIR) at 30 min of OGTT for the novel genetic variant *GRB10* rs933360 (A) and the previously reported T2D and glycemic trait variants *HHEX/IDE/KIF11* (B), *MTNR1B* (C), *CDKAL1* (D), *C2CD4A (NLF1)* (E), *ANK1* (F), *GCK* (G) and *GIPR* (H).
(TIFF)

Figure S2 Genome-wide quantile-quantile (Q-Q) and Manhattan plots for insulin secretion and action traits analyzed in the present study. Corrected insulin response (CIR) to glucose at 30 min of OGTT (A, B), insulin sensitivity index (C, D), insulin response adjusted for insulin sensitivity (CIR adj ISI) (E, F), disposition index (G, H), insulin level at 30 min of OGTT (I, J), incremental insulin at 30 min of OGTT (K, L), insulin level at 30 min of OGTT adjusted for BMI (M, N), area under the insulin curve (AUC ins) (O, P) and ratio between area under the insulin and glucose curves (AUC ins/AUC gluc) (Q, R).
(TIFF)

Figure S3 Meta-analysis for association of *GRB10* rs933360 with fasting plasma glucose in the participating cohorts. The insulin-reducing allele was associated with lower fasting plasma glucose levels in all individuals.
(TIFF)

Figure S4 *GRB10* mRNA levels in human islets. (A) *GRB10* expression levels did not significantly differ between normoglycemic (HbA1c <5.4%) and hyperglycemic (HbA1c >6%) islet donors (T-test, *p = *0.32). (B) There was no significant difference in *GRB10* mRNA expression levels between carriers of different *GRB10* genotypes (linear regression, *p*_normoglycemic_ = 0.11; *p*_hyperglycemic_ = 0.25). (C) Correlation between *GRB10* and *GCG* (glucagon), and *GRB10* and *INS* (insulin) mRNA expression in islets from normoglycemic (HbA1c <6%, N = 51) and hyperglycemic donors (HbA1c >6%, N = 27). Error bars denote SE.
(TIFF)

Figure S5 Schematic representation of the *GRB10* gene and its transcripts. The 5′ exon arrangements of *GRB10* splice variants were identified in human fat and islet samples by RT-PCR and Sanger sequencing. The exon order at the gene level is shown in the top row. UCSC IDs of matching transcripts are indicated to the right.
(TIFF)

Figure S6 Allele-specific expression analysis of the *GRB10* 5′-end in islet, muscle, liver and fat tissue. Transcripts containing exon UN2 are exclusively expressed from one allele (presumably paternal), whereas transcripts containing the upstream exons UN1 or UN1a derive from both alleles to a varying degree. UN1a, UN1 and UN2 are mutually exclusive exons. A sequence of heterozygous genomic DNA (gDNA) is shown on top for comparison (50%-50%). rs1800504 in exon 3 was used as a reporter SNP for the intronic index SNP rs933360.
(TIFF)

Figure S7 Pictorial representation of *GRB10* methylation status in human islets and peripheral blood lymphocytes. The impact of rs933360 on DNA methylation of three CpG sites analyzed using EpiTYPER in human islets (N = 96) (A) and peripheral blood lymphocytes (PBL, N = 18) (B). (C) The *GRB10* mRNA levels correlated negatively with the degree of methylation at CpG sites located 8,196 bp downstream of rs933360 of the *GRB10* gene in human pancreatic islets (N = 66). (D) Methylation status was tested for 44 CpG sites using the Illumina Infinium 450 K global methylation assay and is represented in the graph above the gene structure. The numbers on the Y-axis on the right indicate the mean β values showing methylation status. The colored boxes represent the exons of the four isoforms annotated in the NCBI. Arrows indicate position of the SNPs tested for association. Distances between nearest CpG site tested and: (i) rs6943153 = 6.9 kb, (ii) rs933360 = 8.2 kb and (iii) rs2237457 = 13.8 kb. Hapmap does not show the linkage of the tested SNPs with any of the SNPs tested for methylation status. The CpG sites in the proximity were methylated (β>0.8). *GRB10* is presented on a reverse strand.
(TIFF)

Figure S8 Effect of *GRB10* disruption on islet function and cell survival in human pancreatic islets. (A) *GRB10* knock-down showed reduced glucagon secretion at 1 mM glucose, particularly stronger for forskolin- and K^+^-stimulated glucagon secretion. Reduced *GRB10* expression in human pancreatic islets had modest effects on insulin secretion, i.e. a slight increase in insulin levels at 1 mM glucose. N_insulin_ = 4 and N_glucagon_ = 3 donors of human pancreatic islets; up to 6 measurements in each experiment for each donor. (B) Effect of *GRB10* disruption on insulin and glucagon content (1 non-diabetic donor, 6 measurements in each experiment). (C) *GRB10* knock-down resulted in a reduction of the number of viable cells in human pancreatic islets. (D) Effect of *GRB10* disruption on caspase-3 mRNA expression (2 non-diabetic donors, 3 measurements in each experiment). * *p*<0.05, *** *p*<0.001.
(TIFF)

Table S1 (A) Characteristics of cohorts and study details of analysis metrics and methods. (B) Heritability estimates of parameters of insulin secretion and action indices used in the study. (C) Spearman correlations between insulin secretion and action indices for the non-diabetic participants of the Botnia-PPP study.
(XLS)

Table S2 (A) Association results of selected SNPs from the discovery GWAS, the CardioMetabochip and the *de novo* replication genotyping. (B) Meta-analysis of GWAS and CardioMetabochip studies: association results with parameters of insulin secretion and insulin action during OGTT for loci previously associated with type 2 diabetes (T2D), 2 hour plasma glucose during OGTT (2hPG), fasting plasma glucose (FGlu), fasting insulin (FIns) and obesity.
(XLS)

Table S3 (A) Assessing transmissions of *GRB10* rs933360 and rs6943153 alleles in the Trios from Finland and Sweden. (B) Parent-of-origin effect of *GRB10* rs933360 on parameters of insulin secretion and insulin action, and glucose levels during OGTT in the non-diabetic offspring from the Trio cohorts.
(XLS)

Table S4 Odds ratios for *GRB10* rs933360 for risk of T2D in the MPP study.
(XLS)

Table S5 (A) Descriptive statistics for the Botnia-PPP cohort. (B) Association between *GRB10* rs933360 and glucagon levels in the Botnia-PPP cohort.
(XLS)

Table S6 *GRB10* SNPs associated with fasting and 2 hr glucose levels in 361 non-diabetic individuals from the DGI GWAS study.
(XLS)

Table S7 Measurements of allelic imbalance in the 8 heterozygous carriers of *GRB10* rs1800504 in human islets.
(XLS)

## References

[1] Diabetes Genetics Initiative of Broad Institute of Harvard and MIT and Novartis Institutes of BioMedical Research, Lund University (2007) Saxena R, Voight BF, Lyssenko V, Burtt NP, et al (2007) Genome-Wide Association Analysis Identifies Loci for Type 2 Diabetes and Triglyceride Levels. Science 316: 1331–1336 doi:10.1126/science.1142358 17463246 10.1126/science.1142358

[2] Dupuis J, Langenberg C, Prokopenko I, Saxena R, Soranzo N, et al (2010) New genetic loci implicated in fasting glucose homeostasis and their impact on type 2 diabetes risk. Nat Genet 42: 105–116 Available: http://www.nature.com/ng/journal/v42/n2/abs/ng.520.html. 20081858 10.1038/ng.520 PMC3018764

[3] Manning AK, Hivert M-F, Scott RA, Grimsby JL, Bouatia-Naji N, et al (2012) A genome-wide approach accounting for body mass index identifies genetic variants influencing fasting glycemic traits and insulin resistance. Nat Genet 44: 659–669 doi:10.1038/ng.2274 22581228 10.1038/ng.2274 PMC3613127

[4] Morris AP, Voight BF, Teslovich TM, Ferreira T, Segrè AV, et al (2012) Large-scale association analysis provides insights into the genetic architecture and pathophysiology of type 2 diabetes. Nat Genet 44: 981–990 doi:10.1038/ng.2383 22885922 10.1038/ng.2383 PMC3442244

[5] Scott LJ, Mohlke KL, Bonnycastle LL, Willer CJ, Li Y, et al (2007) A Genome-Wide Association Study of Type 2 Diabetes in Finns Detects Multiple Susceptibility Variants. Science 316: 1341–1345 doi:10.1126/science.1142382 17463248 10.1126/science.1142382 PMC3214617

[6] Scott RA, Lagou V, Welch RP, Wheeler E, Montasser ME, et al (2012) Large-scale association analyses identify new loci influencing glycemic traits and provide insight into the underlying biological pathways. Nat Genet 44: 991–1005 doi:10.1038/ng.2385 22885924 10.1038/ng.2385 PMC3433394

[7] Sladek R, Rocheleau G, Rung J, Dina C, Shen L, et al (2007) A genome-wide association study identifies novel risk loci for type 2 diabetes. Nature 445: 881–885 doi:10.1038/nature05616 17293876 10.1038/nature05616

[8] Zeggini E, Weedon MN, Lindgren CM, Frayling TM, Elliott KS, et al (2007) Replication of Genome-Wide Association Signals in UK Samples Reveals Risk Loci for Type 2 Diabetes. Science 316: 1336–1341 doi:10.1126/science.1142364 17463249 10.1126/science.1142364 PMC3772310

[9] Ingelsson E, Langenberg C, Hivert M-F, Prokopenko I, Lyssenko V, et al (2010) Detailed physiologic characterization reveals diverse mechanisms for novel genetic Loci regulating glucose and insulin metabolism in humans. Diabetes 59: 1266–1275 doi:10.2337/db09-1568 20185807 10.2337/db09-1568 PMC2857908

[10] Lyssenko V, Jonsson A, Almgren P, Pulizzi N, Isomaa B, et al (2008) Clinical risk factors, DNA variants, and the development of type 2 diabetes. N Engl J Med 359: 2220–2232. 19020324 10.1056/NEJMoa0801869

[11] Blagitko N, Mergenthaler S, Schulz U, Wollmann H, Craigen W, et al (2000) Human GRB10 is imprinted and expressed from the paternal and maternal allele in a highly tissue- and isoform-specific fashion. Human Molecular Genetics 9: 1587–1595. 10861285 10.1093/hmg/9.11.1587

[12] Monk D, Arnaud P, Frost J, Hills FA, Stanier P, et al (2009) Reciprocal imprinting of human GRB10 in placental trophoblast and brain: evolutionary conservation of reversed allelic expression. Human Molecular Genetics 18: 3066–3074 doi:10.1093/hmg/ddp248 19487367 10.1093/hmg/ddp248

[13] Rampersaud E, Damcott CM, Fu M, Shen H, McArdle P, et al (2007) Identification of Novel Candidate Genes for Type 2 Diabetes From a Genome-Wide Association Scan in the Old Order Amish: Evidence for Replication From Diabetes-Related Quantitative Traits and From Independent Populations. Diabetes 56: 3053–3062 doi:10.2337/db07-0457 17846126 10.2337/db07-0457

[14] Purcell S, Sham P, Daly MJ (2005) Parental phenotypes in family-based association analysis. Am J Hum Genet 76: 249–259 doi:10.1086/427886 15614722 10.1086/427886 PMC1196370

[15] Horvath S, Xu X, Laird NM (2001) The family based association test method: strategies for studying general genotype–phenotype associations. Eur J Hum Genet 9: 301–306 doi:10.1038/sj.ejhg.5200625 11313775 10.1038/sj.ejhg.5200625

[16] Altshuler D, Hirschhorn JN, Klannemark M, Lindgren CM, Vohl MC, et al (2000) The common PPARgamma Pro12Ala polymorphism is associated with decreased risk of type 2 diabetes. Nat Genet 26: 76–80 doi:10.1038/79216 10973253 10.1038/79216

[17] Nilsson PM, Nilsson JA, Berglund G (2006) Population-attributable risk of coronary heart disease risk factors during long-term follow-up: the Malmo Preventive Project. J Intern Med 260: 134–141 doi:10.1111/j.1365-2796.2006.01671.x 16882277 10.1111/j.1365-2796.2006.01671.x

[18] Arnaud P (2003) Conserved methylation imprints in the human and mouse GRB10 genes with divergent allelic expression suggests differential reading of the same mark. Human Molecular Genetics 12: 1005–1019 doi:10.1093/hmg/ddg110 12700169 10.1093/hmg/ddg110

[19] Nitert MD, Dayeh T, Volkov P, Elgzyri T, Hall E, et al (2012) Impact of an exercise intervention on DNA methylation in skeletal muscle from first-degree relatives of patients with type 2 diabetes. Diabetes 61: 3322–3332 doi:10.2337/db11-1653 23028138 10.2337/db11-1653 PMC3501844

[20] Mootha VK, Lindgren CM, Eriksson K-F, Subramanian A, Sihag S, et al (2003) PGC-1alpha-responsive genes involved in oxidative phosphorylation are coordinately downregulated in human diabetes. Nat Genet 34: 267–273 doi:10.1038/ng1180 12808457 10.1038/ng1180

[21] Stančáková A, Civelek M, Saleem NK, Soininen P, Kangas AJ, et al (2012) Hyperglycemia and a common variant of GCKR are associated with the levels of eight amino acids in 9,369 Finnish men. Diabetes 61: 1895–1902 doi:10.2337/db11-1378 22553379 10.2337/db11-1378 PMC3379649

[22] Doiron B, Hu W, Norton L, DeFronzo RA (2012) Lentivirus shRNA Grb10 targeting the pancreas induces apoptosis and improved glucose tolerance due to decreased plasma glucagon levels. Diabetologia 55: 719–728 doi:10.1007/s00125-011-2414-z 22222503 10.1007/s00125-011-2414-z

[23] Zhang J, Zhang N, Liu M, Li X, Zhou L, et al (2012) Disruption of Growth Factor Receptor-Binding Protein 10 in the Pancreas Enhances β-Cell Proliferation and Protects Mice From Streptozotocin-Induced β-Cell Apoptosis. Diabetes 61: 3189–3198 doi:10.2337/db12-0249 22923474 10.2337/db12-0249 PMC3501856

[24] Holt L, Siddle K (2005) Grb10 and Grb14: enigmatic regulators of insulin action - and more? Biochem J 388: 393–406 doi:10.1042/BJ20050216 15901248 10.1042/BJ20050216 PMC1138946

[25] Yu Y, Yoon S-O, Poulogiannis G, Yang Q, Ma XM, et al (2011) Phosphoproteomic analysis identifies Grb10 as an mTORC1 substrate that negatively regulates insulin signaling. Science Signaling 332: 1322–1326 Available: http://eutils.ncbi.nlm.nih.gov/entrez/eutils/elink.fcgi?dbfrom=pubmed&id=21659605&retmode=ref&cmd=prlinks. 10.1126/science.1199484 PMC3195509 21659605

[26] Wang L, Balas B, Christ-Roberts CY, Kim RY, Ramos FJ, et al (2007) Peripheral disruption of the grb10 gene enhances insulin signaling and sensitivity in vivo. Molecular and Cellular Biology 27: 6497–6505 Available: http://mcb.asm.org/cgi/doi/10.1128/MCB.00679-07. 17620412 10.1128/MCB.00679-07 PMC2099625

[27] Voight BF, Scott LJ, Steinthorsdottir V, Morris AP, Dina C, et al (2010) Twelve type 2 diabetes susceptibility loci identified through large-scale association analysis. Nat Genet 42: 579–589 doi:10.1038/ng.609 20581827 10.1038/ng.609 PMC3080658

[28] Matthews JNS, Altman DG, Campbell MJ, Royston P (1990) Analysis of Serial Measurements in Medical-Research. Brit Med J 300: 230–235 Available: http://www.ncbi.nlm.nih.gov/pmc/articles/PMC1662068/pdf/bmj00163-0030.pdf. 2106931 10.1136/bmj.300.6719.230 PMC1662068

[29] Marchini J, Howie B, Myers S, McVean G, Donnelly P (2007) A new multipoint method for genome-wide association studies by imputation of genotypes. Nat Genet 39: 906–913 doi:10.1038/ng2088 17572673 10.1038/ng2088

[30] Li Y, Willer C, Sanna S, Abecasis G (2009) Genotype imputation. Annu Rev Genomics Hum Genet 10: 387–406 doi:10.1146/annurev.genom.9.081307.164242 19715440 10.1146/annurev.genom.9.081307.164242 PMC2925172

[31] Li Y, Willer CJ, Ding J, Scheet P, Abecasis GR (2010) MaCH: using sequence and genotype data to estimate haplotypes and unobserved genotypes. Genet Epidemiol 34: 816–834 doi:10.1002/gepi.20533 21058334 10.1002/gepi.20533 PMC3175618

[32] Aulchenko YS, Ripke S, Isaacs A, van Duijn CM (2007) GenABEL: an R library for genome-wide association analysis. 10.1093/bioinformatics/btm108 17384015

[33] Devlin B, Roeder K (2004) Genomic Control for Association Studies. Biometrics 55: 997–1004 doi:10.1111/j.0006-341X.1999.00997.x 11315092 10.1111/j.0006-341x.1999.00997.x

[34] Mägi R, Morris AP (2010) GWAMA: software for genome-wide association meta-analysis. BMC Bioinformatics 11: 288 doi:10.1186/1471-2105-11-288 20509871 10.1186/1471-2105-11-288 PMC2893603

[35] Purcell S, Neale B, Todd-Brown K, Thomas L, Ferreira MAR, et al (2007) PLINK: A Tool Set for Whole-Genome Association and Population-Based Linkage Analyses. The American Journal of Human Genetics 81: 559–575 doi:10.1086/519795 17701901 10.1086/519795 PMC1950838

[36] Isomaa B, Forsén B, Lahti K, Holmström N, Wadén J, et al (2010) A family history of diabetes is associated with reduced physical fitness in the Prevalence, Prediction and Prevention of Diabetes (PPP)–Botnia study. Diabetologia 53: 1709–1713 doi:10.1007/s00125-010-1776-y 20454776 10.1007/s00125-010-1776-y

[37] Eriksson KF, Lindgarde F (1990) Impaired glucose tolerance in a middle-aged male urban population: a new approach for identifying high-risk cases. Diabetologia 33: 526–531. 2253828 10.1007/BF00404139

[38] Eriksson KF, Lindgarde F (1991) Prevention of type 2 (non-insulin-dependent) diabetes mellitus by diet and physical exercise. The 6-year Malmö feasibility study. Diabetologia 34: 891–898. 1778354 10.1007/BF00400196

[39] Taneera J, Lang S, Sharma A, Fadista J, Zhou Y, et al (2012) A systems genetics approach identifies genes and pathways for type 2 diabetes in human islets. Cell Metabolism 16: 122–134 doi:10.1016/j.cmet.2012.06.006 22768844 10.1016/j.cmet.2012.06.006

[40] Riva M, Nitert MD, Voss U, Sathanoori R, Lindqvist A, et al (2011) Nesfatin-1 stimulates glucagon and insulin secretion and beta cell NUCB2 is reduced in human type 2 diabetic subjects. Cell Tissue Res 346: 393–405 doi:10.1007/s00441-011-1268-5 22108805 10.1007/s00441-011-1268-5

[41] Pruim RJ, Welch RP, Sanna S, Teslovich TM, Chines PS, et al (2010) LocusZoom: regional visualization of genome-wide association scan results. Bioinformatics 26: 2336–2337 doi:10.1093/bioinformatics/btq419 20634204 10.1093/bioinformatics/btq419 PMC2935401

