# Supplementary figures and images for: A Central Role for GRB10 in Regulation of Islet Function in Man
Source: PLoS Genet. 2014 Apr 3;10(4):e1004235. doi: 10.1371/journal.pgen.1004235 (PMC3974640; doi:10.1371/journal.pgen.1004235)

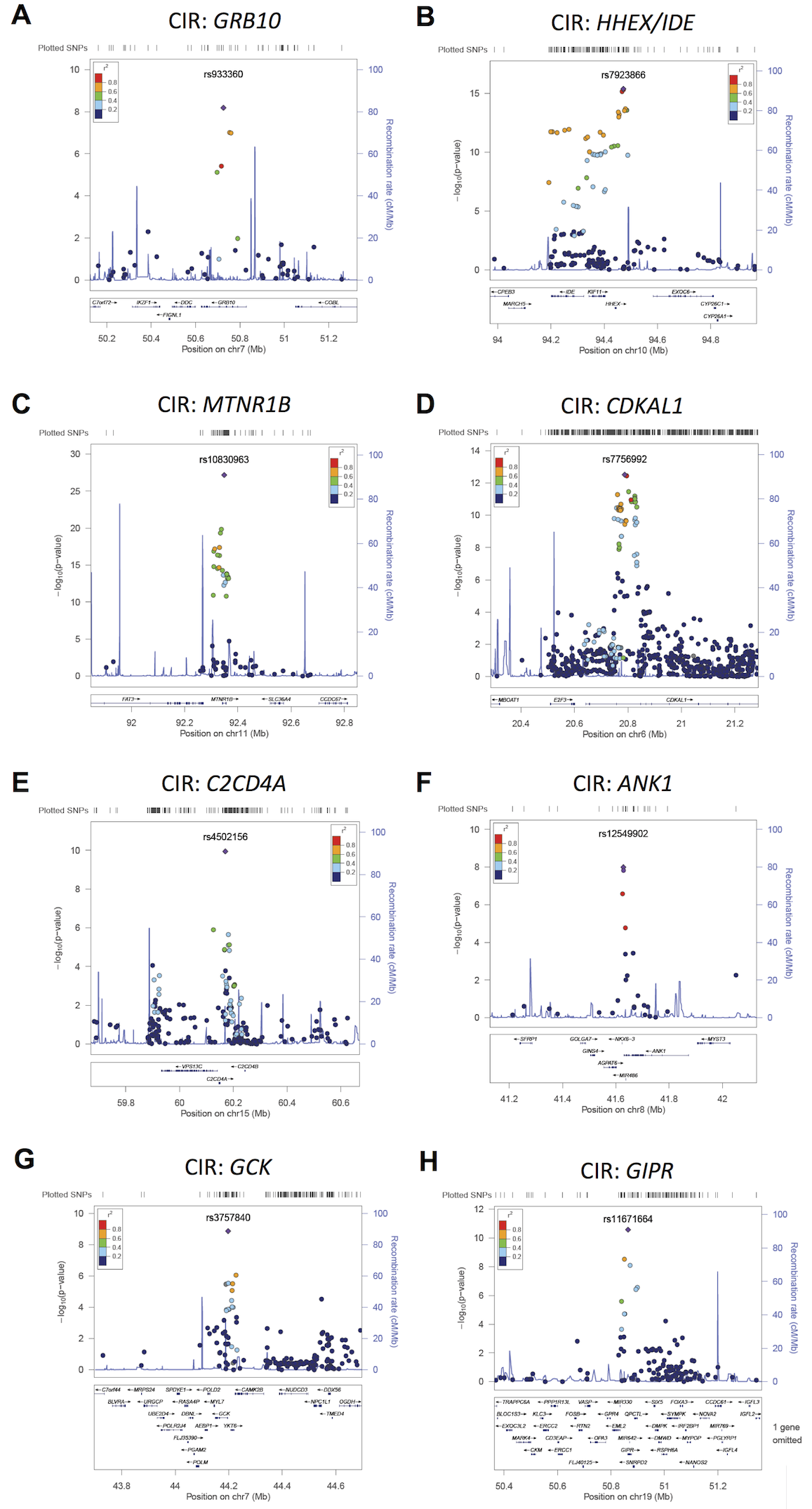

Supplement: Figure S1 — Regional plots for the top 8 hits reaching genome-wide significance level. Association with insulin secretion measured as corrected insulin response (CIR) at 30 min of OGTT for the novel genetic variant GRB10 rs933360 (A) and the previously reported T2D and glycemic trait variants HHEX/IDE/KIF11 (B), MTNR1B (C), CDKAL1 (D), C2CD4A (NLF1) (E), ANK1 (F), GCK (G) and GIPR (H). (TIFF) [file pgen.1004235.s001.tiff]

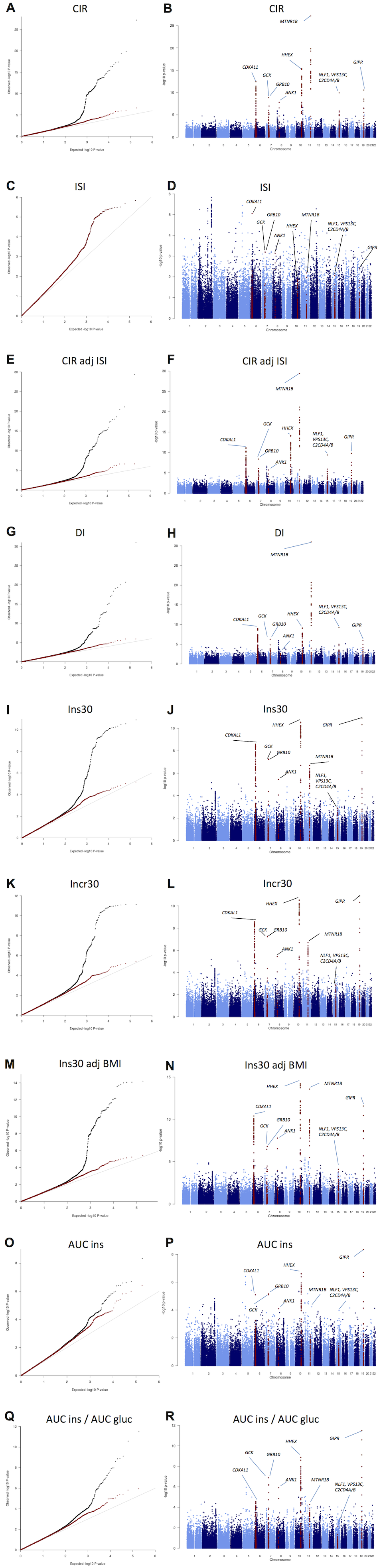

Supplement: Figure S2 — Genome-wide quantile-quantile (Q-Q) and Manhattan plots for insulin secretion and action traits analyzed in the present study. Corrected insulin response (CIR) to glucose at 30 min of OGTT (A, B), insulin sensitivity index (C, D), insulin response adjusted for insulin sensitivity (CIR adj ISI) (E, F), disposition index (G, H), insulin level at 30 min of OGTT (I, J), incremental insulin at 30 min of OGTT (K, L), insulin level at 30 min of OGTT adjusted for BMI (M, N), area under the insulin curve (AUC ins) (O, P) and ratio between area under the insulin and glucose curves (AUC ins/AUC gluc) (Q, R). (TIFF) [file pgen.1004235.s002.tiff]

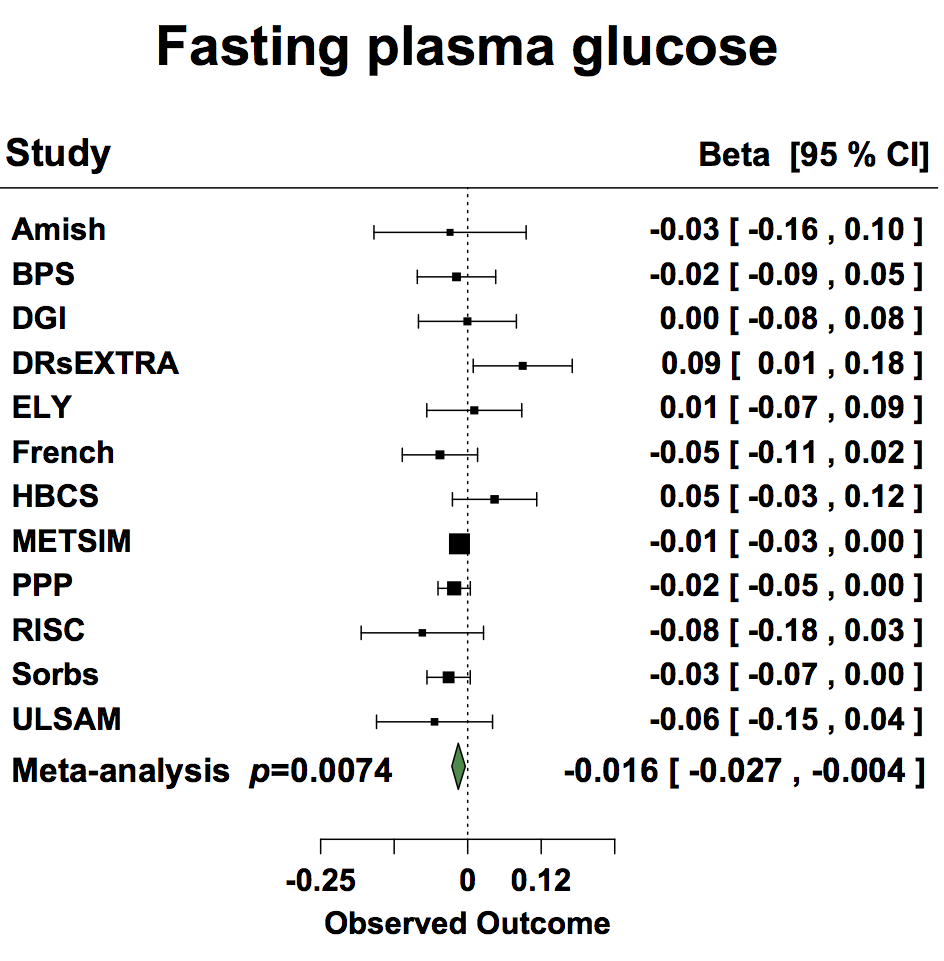

Supplement: Figure S3 — Meta-analysis for association of GRB10 rs933360 with fasting plasma glucose in the participating cohorts. The insulin-reducing allele was associated with lower fasting plasma glucose levels in all individuals. (TIFF) [file pgen.1004235.s003.tiff]

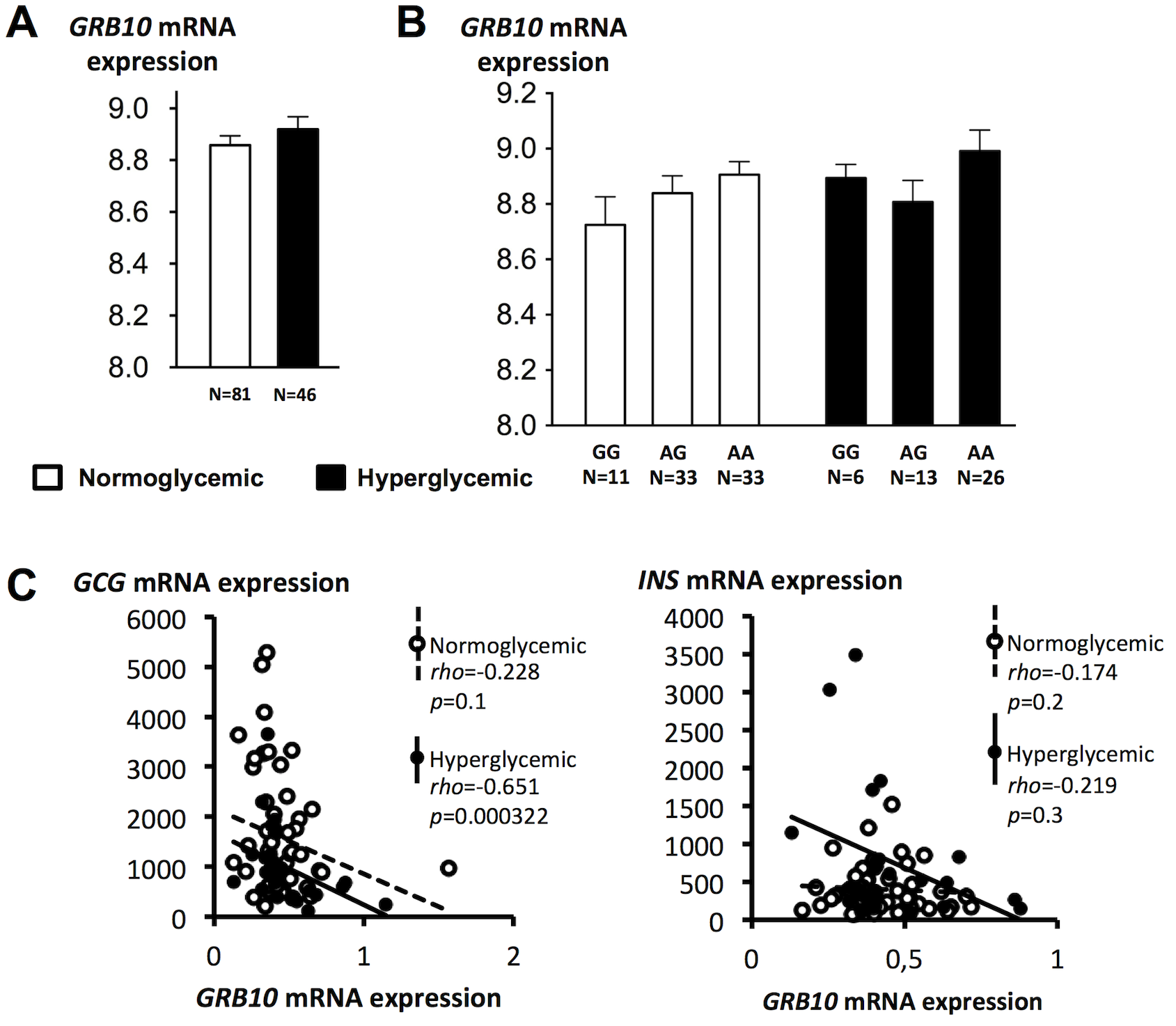

Supplement: Figure S4 — GRB10 mRNA levels in human islets. (A) GRB10 expression levels did not significantly differ between normoglycemic (HbA1c <5.4%) and hyperglycemic (HbA1c >6%) islet donors (T-test, p = 0.32). (B) There was no significant difference in GRB10 mRNA expression levels between carriers of different GRB10 genotypes (linear regression, p normoglycemic = 0.11; p hyperglycemic = 0.25). (C) Correlation between GRB10 and GCG (glucagon), and GRB10 and INS (insulin) mRNA expression in islets from normoglycemic (HbA1c <6%, N = 51) and hyperglycemic donors (HbA1c >6%, N = 27). Error bars denote SE. (TIFF) [file pgen.1004235.s004.tiff]

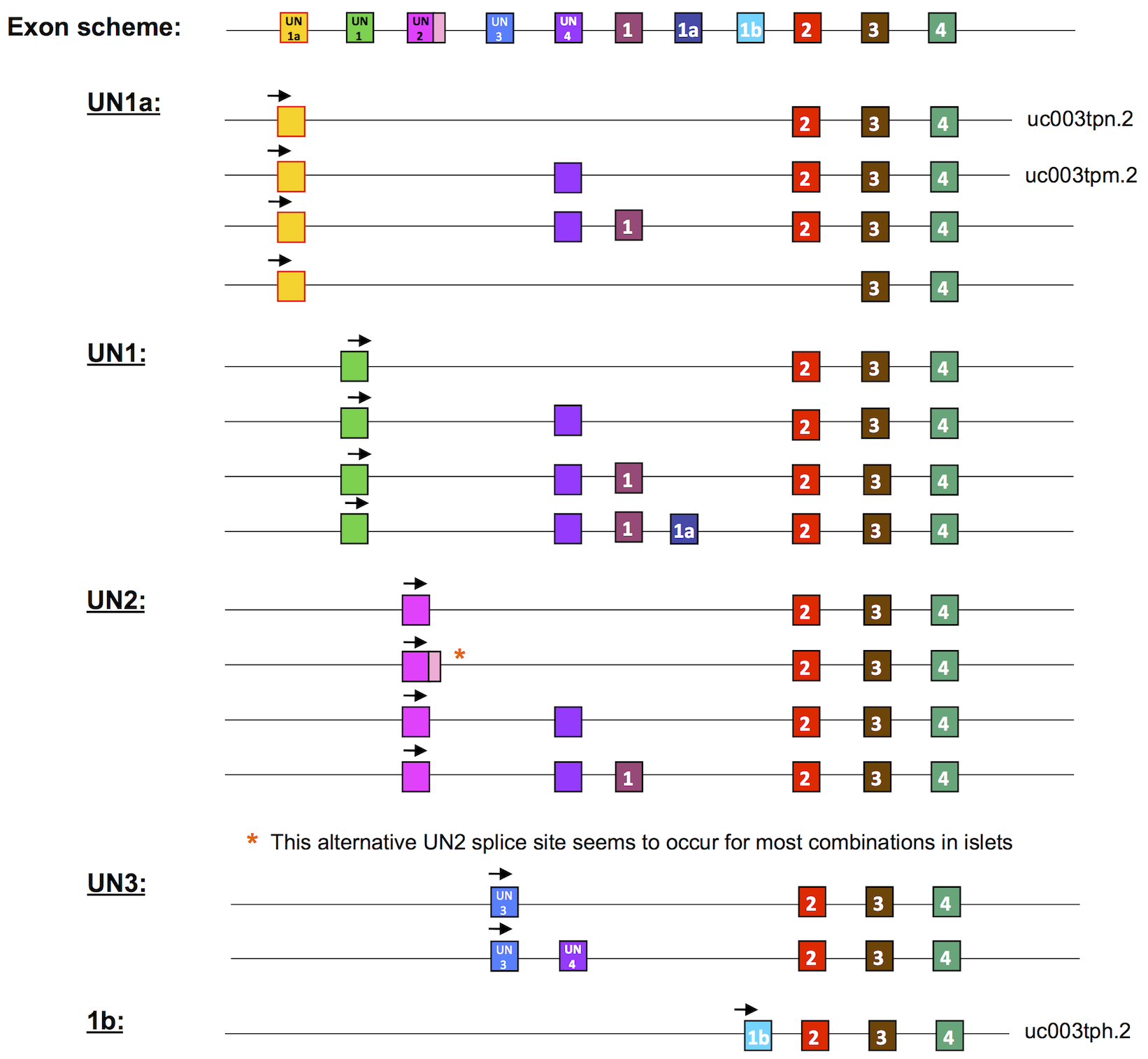

Supplement: Figure S5 — Schematic representation of the GRB10 gene and its transcripts. The 5′ exon arrangements of GRB10 splice variants were identified in human fat and islet samples by RT-PCR and Sanger sequencing. The exon order at the gene level is shown in the top row. UCSC IDs of matching transcripts are indicated to the right. (TIFF) [file pgen.1004235.s005.tiff]

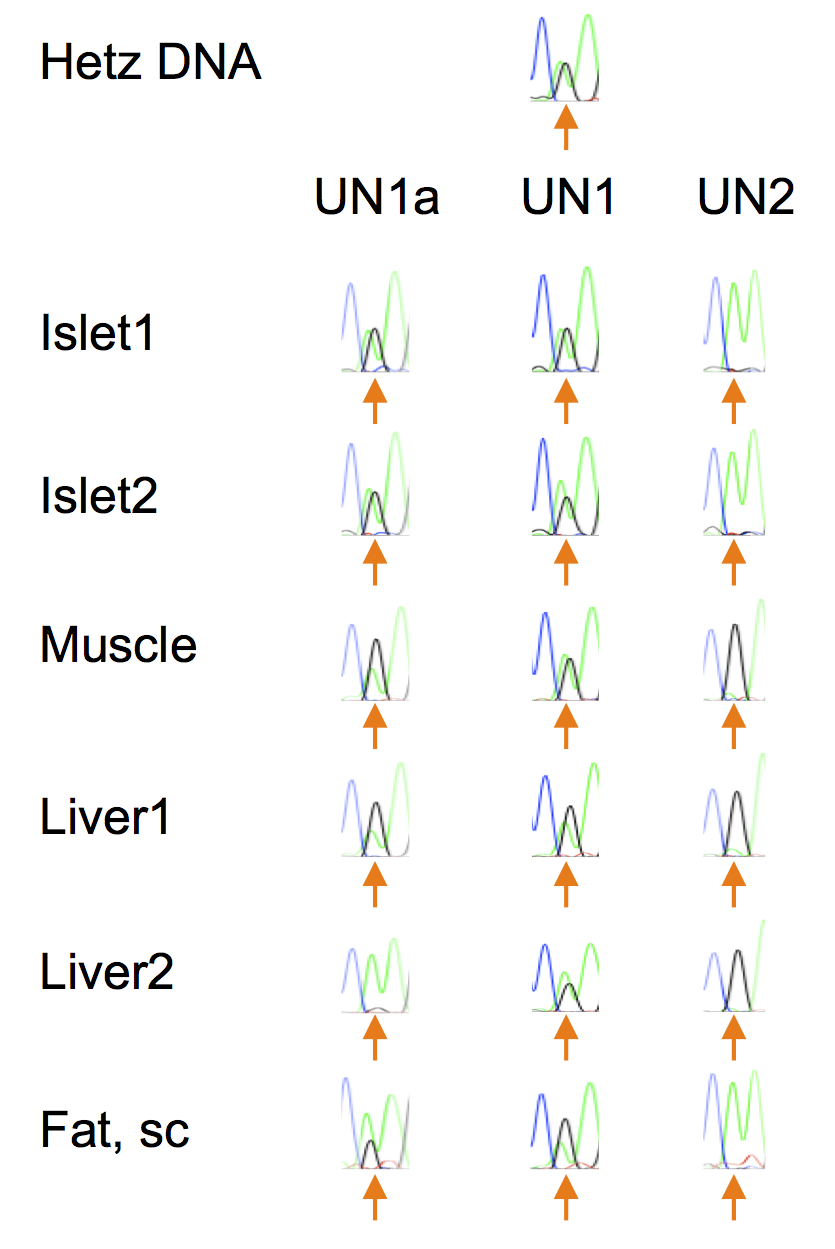

Supplement: Figure S6 — Allele-specific expression analysis of the GRB10 5′-end in islet, muscle, liver and fat tissue. Transcripts containing exon UN2 are exclusively expressed from one allele (presumably paternal), whereas transcripts containing the upstream exons UN1 or UN1a derive from both alleles to a varying degree. UN1a, UN1 and UN2 are mutually exclusive exons. A sequence of heterozygous genomic DNA (gDNA) is shown on top for comparison (50%-50%). rs1800504 in exon 3 was used as a reporter SNP for the intronic index SNP rs933360. (TIFF) [file pgen.1004235.s006.tiff]

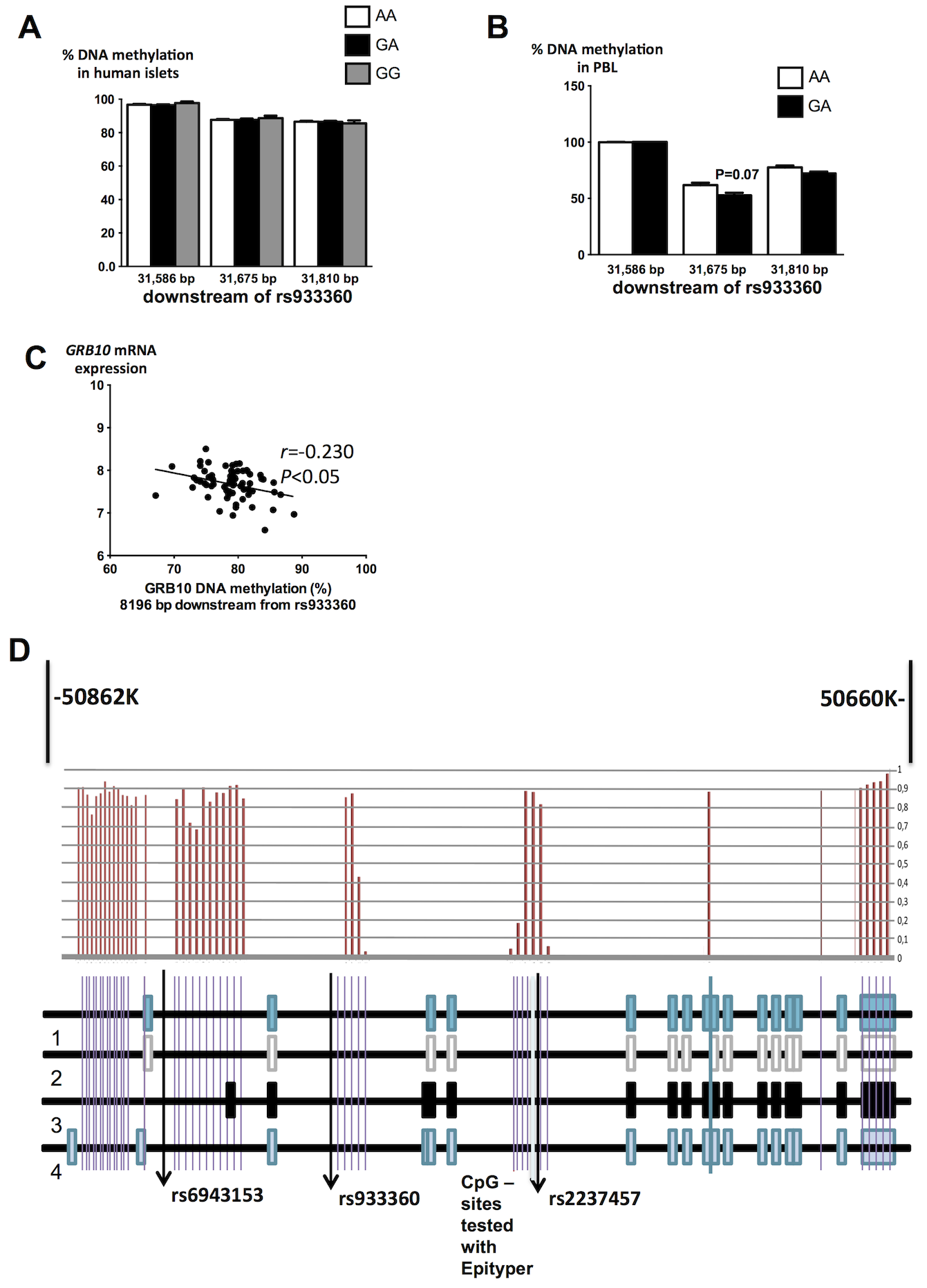

Supplement: Figure S7 — Pictorial representation of GRB10 methylation status in human islets and peripheral blood lymphocytes. The impact of rs933360 on DNA methylation of three CpG sites analyzed using EpiTYPER in human islets (N = 96) (A) and peripheral blood lymphocytes (PBL, N = 18) (B). (C) The GRB10 mRNA levels correlated negatively with the degree of methylation at CpG sites located 8,196 bp downstream of rs933360 of the GRB10 gene in human pancreatic islets (N = 66). (D) Methylation status was tested for 44 CpG sites using the Illumina Infinium 450 K global methylation assay and is represented in the graph above the gene structure. The numbers on the Y-axis on the right indicate the mean β values showing methylation status. The colored boxes represent the exons of the four isoforms annotated in the NCBI. Arrows indicate position of the SNPs tested for association. Distances between nearest CpG site tested and: (i) rs6943153 = 6.9 kb, (ii) rs933360 = 8.2 kb and (iii) rs2237457 = 13.8 kb. Hapmap does not show the linkage of the tested SNPs with any of the SNPs tested for methylation status. The CpG sites in the proximity were methylated (β>0.8). GRB10 is presented on a reverse strand. (TIFF) [file pgen.1004235.s007.tiff]

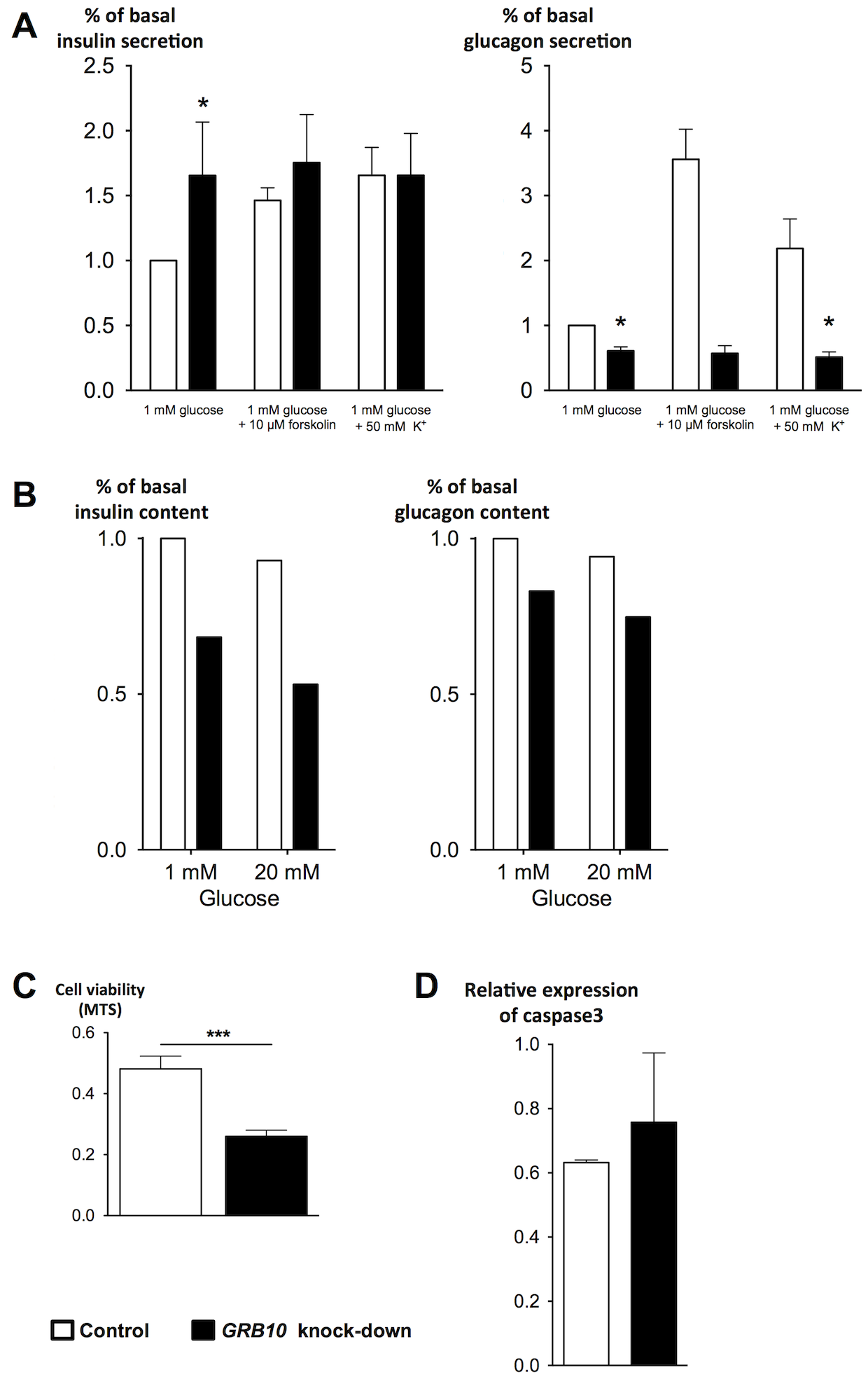

Supplement: Figure S8 — Effect of GRB10 disruption on islet function and cell survival in human pancreatic islets. (A) GRB10 knock-down showed reduced glucagon secretion at 1 mM glucose, particularly stronger for forskolin- and K+-stimulated glucagon secretion. Reduced GRB10 expression in human pancreatic islets had modest effects on insulin secretion, i.e. a slight increase in insulin levels at 1 mM glucose. Ninsulin = 4 and Nglucagon = 3 donors of human pancreatic islets; up to 6 measurements in each experiment for each donor. (B) Effect of GRB10 disruption on insulin and glucagon content (1 non-diabetic donor, 6 measurements in each experiment). (C) GRB10 knock-down resulted in a reduction of the number of viable cells in human pancreatic islets. (D) Effect of GRB10 disruption on caspase-3 mRNA expression (2 non-diabetic donors, 3 measurements in each experiment). * p<0.05, *** p<0.001. (TIFF) [file pgen.1004235.s008.tiff]
